# Potent, Reversible, and Specific Chemical Inhibitors of Eukaryotic Ribosome Biogenesis

**DOI:** 10.1016/j.cell.2016.08.070

**Published:** 2016-10-06

**Authors:** Shigehiro A. Kawashima, Zhen Chen, Yuki Aoi, Anupam Patgiri, Yuki Kobayashi, Paul Nurse, Tarun M. Kapoor

**Affiliations:** 1 The University of Tokyo, Graduate School of Pharmaceutical Sciences, Tokyo 1130033, Japan; 2 Laboratory of Chemistry and Cell Biology, The Rockefeller University, New York, NY 10065, USA; 3 Laboratory of Yeast Genetics and Cell Biology, The Rockefeller University, New York, NY 10065, USA; 4 The Francis Crick Institute, London NW1 2BE, UK

**Keywords:** ribosome biogenesis, chemical inhibitors, AAA^+^ protein, Midasin, chemical genetics, chemical screen, fission yeast, *Schizosaccharomyces pombe*

## Abstract

All cellular proteins are synthesized by ribosomes, whose biogenesis in eukaryotes is a complex multi-step process completed within minutes. Several chemical inhibitors of ribosome function are available and used as tools or drugs. By contrast, we lack potent validated chemical probes to analyze the dynamics of eukaryotic ribosome assembly. Here, we combine chemical and genetic approaches to discover ribozinoindoles (or Rbins), potent and reversible triazinoindole-based inhibitors of eukaryotic ribosome biogenesis. Analyses of Rbin sensitivity and resistance conferring mutations in fission yeast, along with biochemical assays with recombinant proteins, provide evidence that Rbins’ physiological target is Midasin, an essential ∼540-kDa AAA+ (ATPases associated with diverse cellular activities) protein. Using Rbins to acutely inhibit or activate Midasin function, in parallel experiments with inhibitor-sensitive or inhibitor-resistant cells, we uncover Midasin’s role in assembling Nsa1 particles, nucleolar precursors of the 60S subunit. Together, our findings demonstrate that Rbins are powerful probes for eukaryotic ribosome assembly.

## Highlights

• Ribozinoindoles are potent chemical inhibitors of eukaryotic ribosome assembly
• Activity of four of Mdn1’s six ATPase sites is likely needed for cell growth
• Ribozinoindoles inhibit recombinant full-length Mdn1’s ATPase activity in vitro
• Assembly of Nsa1 particles, precursors of the 60S subunit, depends on Mdn1

## Graphical Abstract

**Figure:**
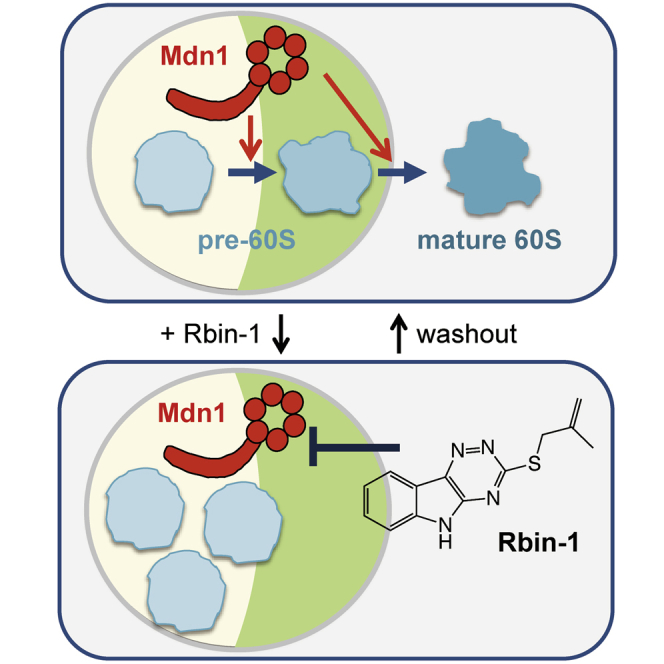

## Teaser

Selective potent inhibitors of eukaryotic ribosome biogenesis are found through a chemical synthetic lethal screen.

## Introduction

Every minute a growing cell can generate ∼2,000 ribosomes, ribonucleoprotein-based nanomachines that translate information encoded in the genome to synthesize proteins (Thomson et al., 2013, Tschochner and Hurt, 2003, Warner, 1999). The assembly of ribosomes, which contain ∼5,500 nucleotides of RNA and ∼80 proteins that are together organized into the 60S and 40S subunits, starts in the nucleolus with the transcription of rDNA. Subsequent steps lead to the processing of RNA to generate 25S, 5.8S, and 5S rRNAs (ribosomal RNAs) for the 60S subunit and the 18S rRNA for the 40S subunit. These steps are coupled to the ordered association and dissociation of several different ribosomal and non-ribosomal proteins to form pre-ribosomal subunits that accumulate in the nucleus. Properly assembled precursors are then exported to the cytoplasm, where the final processing steps lead to functional ribosomes (Thomson et al., 2013, Tschochner and Hurt, 2003). Currently, it is unclear how such elaborate spatial and temporal coordination of the several steps required for ribosome assembly is achieved by ∼200 non-ribosomal proteins (Thomson et al., 2013, Tschochner and Hurt, 2003). This is in large part due to the lack of tools and approaches to analyze this essential and dynamic cellular process.

The assembly of the 60S subunit involves multiple intermediates, collectively referred to as pre-60S particles (Nissan et al., 2002). The rearrangements of these particles have been characterized using imaging and immunoprecipitation-based approaches, and the key intermediates have been linked to specific proteins that associate with distinct particles, such as the Nsa1-associated complex (named the Nsa1 particles) assembled in the nucleolus and the Rix1-associated complex (named the Rix1 particles) processed in the nucleoplasm (Kressler et al., 2012). The fidelity of this dynamic assembly process depends on precise spatial and temporal regulation at each stage. For example, a Nug2 GTPase-dependent checkpoint blocks export of pre-60S particles to the cytoplasm until it is properly assembled (Matsuo et al., 2014). The sequential processing of these ribosome assembly intermediates requires numerous energy-consuming enzymes, including ATPases in the AAA+ (ATPases associated with diverse cellular activities) family (Kressler et al., 2012, Thomson et al., 2013).

The AAA+ family is a large and functionally diverse group of enzymes that can couple ATP hydrolysis with mechanical work (e.g., protein unfolding or directional transport) (Erzberger and Berger, 2006, Hanson and Whiteheart, 2005). At least three AAA+ ATPases, Drg1, Rix7, and Midasin (Rea1 in *S. cerevisiae* and Mdn1 in *S. pombe*), are involved in the assembly of the 60S subunit (Kressler et al., 2012). Drg1 and Rix7 are closely related to the well-studied Cdc48 (p97/VCP in mammals) and likely function as homohexamers with six equivalent ATPase sites (Patel and Latterich, 1998). By contrast, Midasin has all six ATPase domains within a single long polypeptide, an organization that is similar to that of the microtubule-based motor protein cytoplasmic dynein (Kressler et al., 2012). Midasin’s ATPase domains form a ring-like domain from which extends an elongated “tail” with a Metal Ion-Dependent Adhesion Site (MIDAS) domain at its end (Garbarino and Gibbons, 2002, Ulbrich et al., 2009). Currently, the ATPase activity of Midasin, one of the largest proteins in yeast, has not been characterized, and it is unclear whether all of its ATPase domains are essential for ribosome biogenesis.

Our current models for Midasin function in 60S biogenesis are based in large part on studies of the *S. cerevisiae* ortholog, Rea1. Knockdown of Rea1 in budding yeast leads to the accumulation of pre-60S particles in the nucleus (Galani et al., 2004). Rea1’s nucleoplasmic function has been linked to the Rix1 particles, where Rea1 is enriched (Galani et al., 2004, Nissan et al., 2002, Nissan et al., 2004). Rea1 interacts with Rsa4, another non-ribosomal protein present in the Rix1 particles. Overexpression of an Rsa4 mutant that fails to interact with Rea1 causes dominant-negative defects in 60S biogenesis (Ulbrich et al., 2009). In vitro experiments show that ATP addition can dissociate Rsa4, Rea1, and Rix1 from the Rix1 particles pulled down from wild-type cells, but not from cells overexpressing Rea1 with mutations in its AAA domain or the MIDAS domain (Matsuo et al., 2014, Ulbrich et al., 2009). Rea1 also interacts with Ytm1, a non-ribosomal protein that mainly associates with nucleolar Nsa1 particles, precursors of the Rix1 particles (Bassler et al., 2010). These data, together with additional studies of the Rix1 particles and Rea1 (Ulbrich et al., 2009), have led to a model in which ATP hydrolysis-dependent motion of Rea1’s “tail” leads to dissociation of Rsa4 from nucleoplasmic pre-60S particles and Ytm1 from nucleolar pre-60S particles (Kressler et al., 2012). However, in order to dissect Midasin’s functions in living cells, we need acute inhibition so that we can distinguish between direct effects of Midasin inhibition from cumulative defects resulting from blocking earlier stages of ribosome biogenesis. This is particularly important as conventional genetic analyses, using temperature-sensitive strains or overexpression of dominant-negative mutants, suppress protein function over hours, while many steps of ribosome biogenesis are completed within minutes.

Cell-permeable chemical inhibitors can be powerful tools for examining dynamic cellular processes, such as ribosome biogenesis, as the functions of target proteins can be blocked within minutes. Currently, the only known chemical inhibitor that directly targets eukaryotic ribosome assembly factors is diazaborine, an antibacterial compound active only at ∼0.4 mM in *S. cerevisiae* (Loibl et al., 2014), a concentration at which selective target inhibition may be difficult to achieve. Moreover, because diazaborine blocks cytoplasmic steps (i.e., pre-60S maturation) of ribosome biogenesis, we lack chemical probes for the several distinct assembly steps that occur in the nucleolus and nucleus. Lamotrigine is another chemical inhibitor of ribosome assembly factors that has been recently described (Stokes et al., 2014). However, this compound has been shown to only block ribosome biogenesis in *E. coli* at low temperatures (Stokes et al., 2014).

In this study, we identify ribozinoindoles (or Rbins), as potent, reversible, and specific inhibitors of Midasin. Systematic genetic analyses of Rbins’ sensitivity and RBins’ resistance in fission yeast, along with biochemical characterization of Mdn1’s ATPase activity, indicate that Rbins directly and specifically inhibit Mdn1 function in vitro and in cells. We combine microscopy, biochemical approaches, and the use of Rbins to inhibit or activate Midasin on the timescale of minutes to analyze ribosome assembly dynamics. Our findings uncover a previously uncharacterized function of Midasin in assembling nucleolar Nsa1 particles.

## Results

### Discovery of Rbin-1 Using a “Chemical Synthetic Lethal” Screen

**Figure 1:**
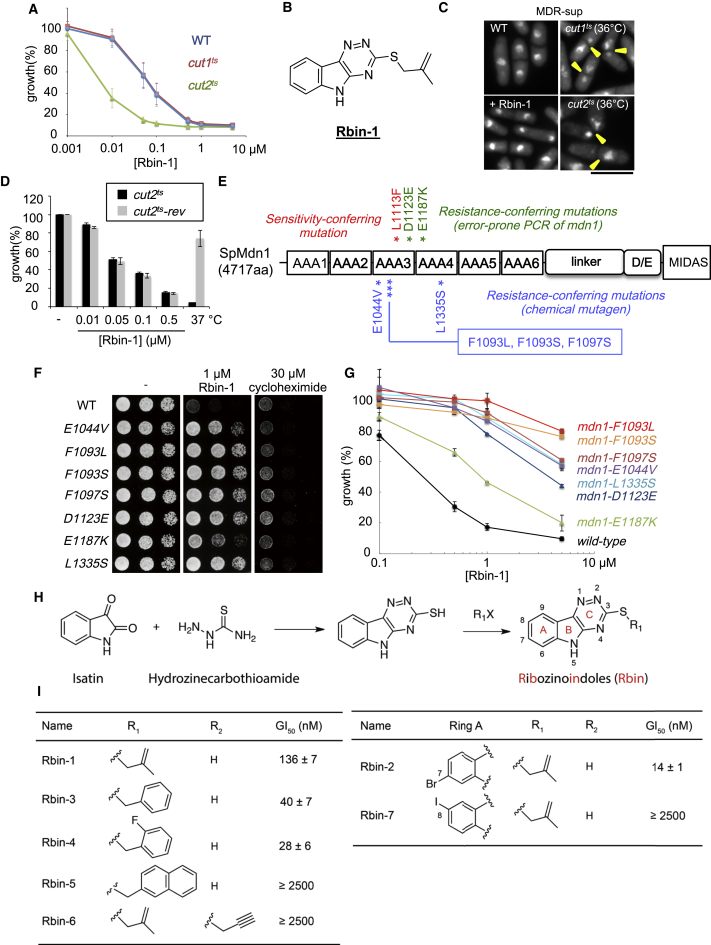
Characterization of Rbin-1, a Potent Chemical Inhibitor of Cell Growth (A) MDR-sup (WT), MDR-sup *cut1-22* (*cut1*^*ts*^), and MDR-sup *cut2-364* (*cut2*^*ts*^) cells were incubated with different concentrations of Rbin-1 (17 hr, 29°C). Growth (%) is presented relative to DMSO-treated cells (mean ± SD, n = 3 independent experiments, trace: interpolation). (B) Chemical structure of Rbin-1. (C) Nuclear morphology of MDR-sup cells (WT), MDR-sup *cut1-22* (*cut1*^*ts*^) and MDR-sup *cut2-364* (*cut2*^*ts*^) cells at 36°C for 2.5 hr (restrictive temperature), and MDR-sup cells treated with 10 μM Rbin-1 at 29°C for 2.5 hr (+Rbin-1) were examined. Representative images are shown. Cells with *cut* phenotype, observed in temperature-sensitive *cut1* or *cut2* mutants, are highlighted (yellow arrowheads). Scale bars, 10 μm. (D) *cut2-364* (*cut2*^*ts*^) and a *cut2*^*ts*^*-rev* strain (with the *cut2-364* mutation reverted to wild-type *cut2*^*+*^) (see also Figure S1B) were incubated (17 hr) with indicated concentrations of Rbin-1 at 29°C or untreated at 37°C. Growth (%) is presented relative to DMSO-treated cells incubated at 29°C (mean ± range, n = 2 independent experiments). (E) Domain organization of the *S. pombe* Mdn1 protein (Sp Mdn1). The AAA domains, linker, Asp/Glu-rich region (D/E) and MIDAS domain are highlighted. Locations of a Rbin-1 sensitivity-conferring mutation (red), Rbin-1 resistance conferring mutations obtained by error-prone PCR of *mdn1* gene (green), and Rbin-1 resistance-conferring mutations obtained using a chemical mutagen (blue) are indicated. (F) Serial dilutions of the indicated *mdn1* mutants were spotted onto YE4S plate, or YE4S plate containing indicated compounds, and incubated at 29°C for 2 days. (G) Wild-type and indicated *mdn1* mutants were incubated for 17 hr at 29°C with indicated concentrations of Rbin-1. Growth (%) is presented relative to DMSO-treated cells (mean ± range, n = 2 independent experiments). (H) General synthesis scheme for RBin analogs (R_1_X: halogenated alkyl/aryl substitutent R_2_: substituent at position-5 of B-ring nitrogen). (I) Activity of Rbin analogs. Wild-type cells were incubated with Rbin analogs (18 hr, 29°C). Half maximum growth inhibition (GI_50_, mean ± SD, n = 3 independent experiments) was determined by fitting relative growth to a four-parameter sigmoidal dose-response curve in PRISM. A summary of all the strains used is provided in Table S1. See also Figures S1 and S2.

**Figure S1:**
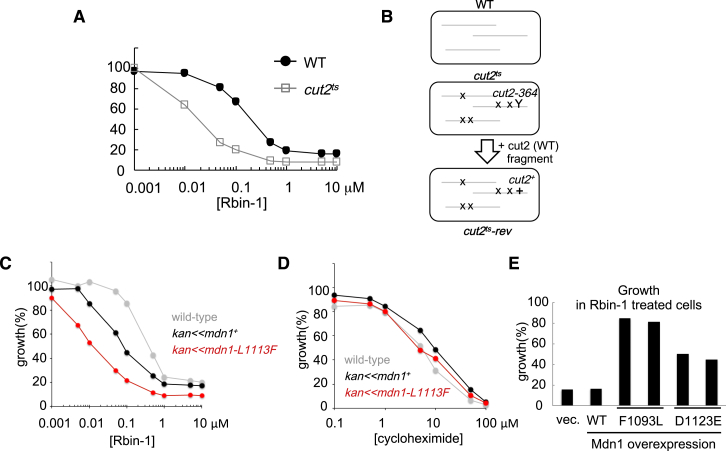
Analysis of Rbin-1-Sensitive and -Resistant *mdn1* Mutants, Related to Figure 1 (A) The *cut2-364* (*cut2*^*ts*^) strain shows higher sensitivity to Rbin-1 than wild-type (WT). Exponentially growing culture (OD = 0.5) of WT (black circle) and *cut2*^*ts*^ (gray square) cells were diluted 50 times in YE4S medium and treated with Rbin-1, and incubated for 17 hr at 29°C. Growth (%) is presented relative to DMSO-treated cells. (B) Schematic shows potential difference between wild-type (WT) and *cut2-364* (*cut2*^*ts*^) strains. X indicates background mutations in *cut2-364* strain. Y indicates a mutation in the *cut2* gene. “*cut2*^*ts*^*-rev*” strain was constructed by utilizing homologous recombination between *cut2-364* mutation and cut2 (WT) fragments. (C and D) A point mutation in the *mdn1* gene (L1113F) confers Rbin-1 sensitivity. Exponentially growing culture (OD = 0.5) of wild-type (square), *kan < < mdn1*^*+*^ (triangle), and *kan < < mdn1 (L1113F)* (circle) cells were diluted 50 times in YE4S medium and treated with RBin-1(C) or cycloheximide (D), and incubated for 17 hr at 29°C. Growth (%) is presented relative to DMSO-treated cells. (E) Overexpression of Mdn1 mutants (F1093L or D1123E) confers Rbin-1 resistance. Exponentially growing culture of MDR-sup strains, in which wild-type (WT) or mutants (F1093L or D1123E) of full-length Mdn1, or vector control (-) were overexpressed (from the nmt1 promoter), were diluted 25 times in EMM-L medium and treated with 1 μM Rbin-1, and incubated for 18.5 hr at 32°C. Growth (%) is presented relative to DMSO-treated cells.

To identify cell-permeable chemical probes of essential cellular processes, we have developed fission yeast as a model system that allows us to efficiently combine genetic and chemical approaches (Aoi et al., 2014, Kawashima et al., 2012). In particular, we have generated fission yeast strains (named “MDR-sup” strains) lacking critical factors for multi-drug resistance (or MDR) and have used them for chemical screens that mimic synthetic lethal genetic screens (Kawashima et al., 2012, Kawashima et al., 2013). We hypothesized that compounds that reveal enhanced toxicity to strains with a particular mutation, compared to a control strain, are likely to be more selective for a single protein target. Consistent with this hypothesis, our use of this strategy identified a selective inhibitor for Aurora kinase, a key cell-cycle regulator (Kawashima et al., 2013). From a similar chemical synthetic lethal screen carried out with a 10,353-member library of diverse chemicals, we identified a triazinoindole-based heterocycle, which we named ribozinoindole-1 (or Rbin-1), that was more toxic to the MDR-sup cells that contained a mutation in *cut2,* compared to those with a wild-type *cut2*^*+*^ or a mutation in *cut1* (Figures 1A and 1B). Both Cut1 and Cut2 are essential proteins needed for faithful chromosome segregation (Funabiki et al., 1996). Subsequent analyses indicated that Rbin-1’s “synthetic lethality” was not due to a chromosome mis-segregation (“*cut*”) phenotype (Figure 1C). In addition, we found that sensitivity to the *cut2* mutant was also observed in strains with the wild-type background (Figure S1A), but reverting the *cut2* mutation to wild-type *cut2*^*+*^ did not suppress Rbin-1 sensitivity (Figures 1D and S1B). Together, these data indicate that the increased sensitivity to the chemical inhibitor was due to other “background” mutations that were likely introduced when the mutant strain was first generated (Hirano et al., 1986).

To examine the mode of action of Rbin-1, we backcrossed the *cut2* mutant MDR-sup strain with wild-type *cut2*^*+*^ MDR-sup and then analyzed the entire genome for mutations. Sequencing analysis identified four genes (SPCC737.08, SPAC14C4.01c, SPAC26A3.01, and SPAC26A3.05) bearing mutations, among which we found that a point mutation (Leu1113Phe) in the *mdn1* (SPCC737.08) gene confers sensitivity to Rbin-1, but not cycloheximide (Figures 1E, S1C, and S1D), suggesting that Rbin-1 may target Mdn1 or cellular processes that involve this protein.

To further address the mode of action of Rbin-1, we chemically mutagenized the MDR-sup fission yeast cells and isolated resistant clones that can grow in the presence of Rbin-1. Each of the 13 Rbin-1 resistant clones contained one of five different point mutations in the *mdn1* gene (Figure 1E). In separate experiments using a PCR-based random mutagenesis strategy, we isolated two additional *mdn1* mutations that confer Rbin-1 resistance (Figure 1E). We next confirmed, using overexpression on plasmids in MDR-sup cells and by replacement of endogenous *mdn1* in wild-type cells, that all the mutations we identified were sufficient to confer resistance to Rbin-1 (Figures 1F, 1G, and S1E). Importantly, these mutations did not confer resistance to cycloheximide, another chemically unrelated inhibitor (Figure 1F). Interestingly, all seven of these resistance-conferring mutations and the sensitivity-conferring mutation clustered around two (AAA3 and AAA4) of the six AAA domains in Mdn1 (Figure 1E). Together, our findings indicate that different mutations in Mdn1 can either enhance or suppress Rbin-1’s activity in both MDR-sup and wild-type cells. Hereafter, for further studies, we use fission yeast strains with the wild-type background rather than the MDR-sup strains.

### Structure-Activity Analyses Lead to a More Potent Rbin-1 Analog

**Figure S2:**
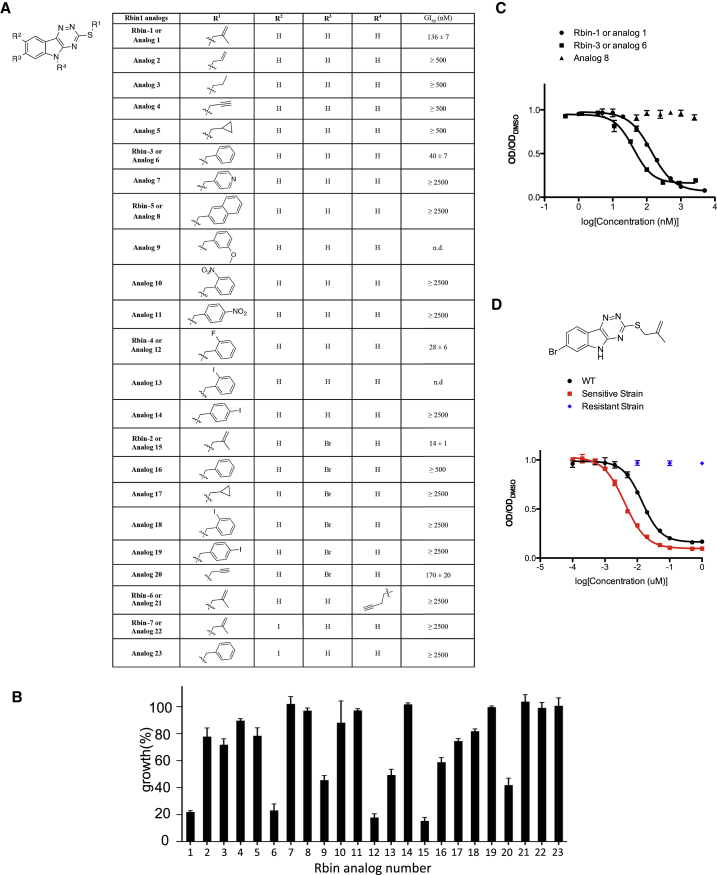
Analysis of Rbin-1 Analogs, Related to Figure 1 (A) Structure-activity relationship of Rbin analogs (summary is provided in Figure 1I). Compounds were added to wild-type cells in diluted culture (in logarithmic phase, OD_600_ ∼0.01) and the mixture was incubated for ∼18 hr at 29°C till OD_600_ = ∼1 for DMSO control. Half maximum growth inhibition (GI_50_, mean ± SD, at least three independent experiments) was determined by fitting relative growth to a four-parameter sigmoidal dose-response curve in PRISM. For compounds that inhibited growth by less than 50% at 500 nM or 2500 nM we indicate GI_50_ to be ” ≥ 500 nM” or ” ≥ 2500 nM” respectively. For some compounds dose-dependent inhibition could not be readily fit to standard equations, likely due to limited compound solubility (indicated as n.d.). (B) Growth inhibition by Rbin analogs at 500 nM. Growth (%) is presented relative to DMSO-treated cells (mean ± SDs, three independent experiments, 500 nM data points for Rbin-1 and RBin-2 are the same as those in panels C and D). (C) Three examples of dose-dependent inhibition of yeast growth by Rbin analogs (mean±SDs, three independent experiments, the curves are fitted by the four-parameter sigmoidal function as in [A]). (D) Rbin-2 is more toxic to the RBin-1-sensitive strain (L1113F mutation in Mdn1) compared to WT cells and significantly less toxic to a Rbin-1-resistant strain (L1093F mutation in Mdn1) (mean±SDs, three independent experiments, the curves are fitted by the four-parameter sigmoidal function as in [A]).

We next optimized the chemical inhibitor’s potency by generating a focused collection of analogs. Twenty-three compounds were synthesized by condensing isatin (or derivatives) with hydrazinecarbothioamide and further elaborated using halogenated aliphatic and aromatic moieties (Figure 1H). This procedure yielded Rbin-1 analogs in >95% purity and 7%–65% overall yield (see Supplemental Information). Dose-dependent analyses were used to determine the potency of these analogs in growth assays (Figure 1I: data for selected compounds; and Figures S2A and S2B: full dataset). Our studies reveal that alkyl substituents at the 3-thiol position result in compounds with activities that are reduced compared to that of Rbin-1 (Figure S2A). A benzyl moiety at the 3-thiol position increased potency but reduced solubility. Larger aromatic substitutions at this position reduced compound activity (Figures 1I and S2). A homo-propargyl modification at the indole nitrogen (R_2_ position) also resulted in an inactive analog (Figure 1I). We found that the most striking changes in activity resulted from modifications of the A-ring (Figure 1I). In particular, an analog (Rbin-2) with a bromine substituent at postion-7 was ∼10-fold more active than Rbin-1 (GI_50_ = 14 ± 1 nM (Rbin-2); 136 ± 7 nM (Rbin-1), n = 4, mean ± SD, Figure 1I). In contrast, an iodine substituent at position-8 suppressed activity (Figure 1I). We confirmed that Rbin-2 activity against the strain expressing the resistance-conferring mutation (*mdn1-F1093L*) was substantially reduced, while activity against the strain expressing the sensitivity-conferring mutation (*mdn1-L1113F*) was increased relative to the wild-type strain (Figure S2D). Together, these tests identify Rbin-2 as a potent Rbin-1 analog that is likely to have a similar mechanism of action.

### At Least Four of the Six ATPase Sites in Mdn1 Are Required for Growth

**Figure 2:**
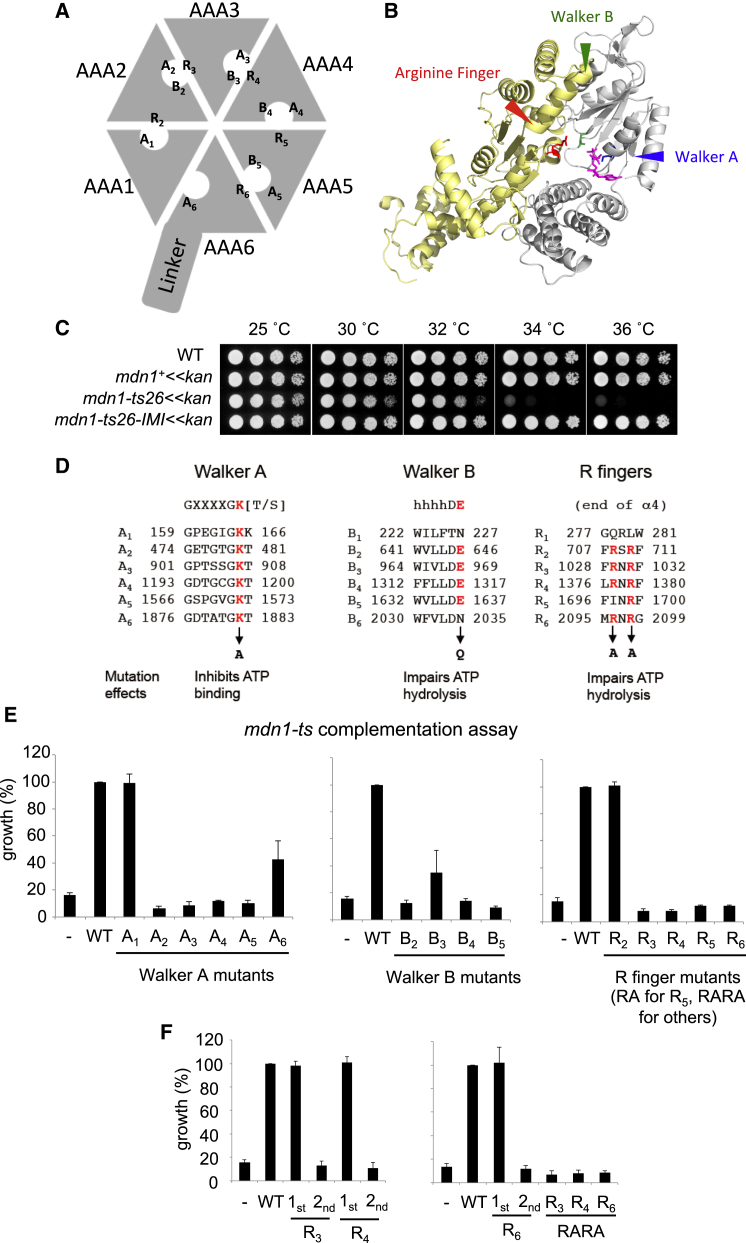
Analyses of the Contributions of Midasin’s ATPase Sites to Cell Growth (A) Schematic for Mdn1’s ATPase domains. The different Walker A (A_n_, n = 1–6), Walker B (B_n_), and arginine finger (R_n_) motifs, which could be readily identified, are indicated. (B) Model for the AAA3 (gray)-AAA4 (yellow) domains of dynein shows the nucleotide-binding site (PDB: 4AKH; AMPPNP: magenta). Selected residues are highlighted (Walker A lysine, blue; Walker B glutamate, green; arginine finger, red). (C) Temperature sensitivity of the indicated strains was examined. (D) Primary sequence for the Walker A, Walker B, and arginine finger motifs from each of Mdn1’s six AAA domains are shown. Residues mutated for experiments in (E) and (F) are highlighted (red). (E and F) Exponentially growing culture of *mdn1-ts26* strains in which wild-type (WT) or indicated mutants of full-length Mdn1 or vector control (–) was overexpressed from the nmt1 promoter in the absence of thiamine were incubated for 24 hr (36°C). Growth (%) is presented relative to WT-overexpressed cells (mean ± SD, n = 4 independent experiments, each bar graph shows data for mutant- or WT-overexpressing cells tested side by side). For R finger mutants, RARA indicates that both arginines in this motif are mutated to alanines, and 1_st_ or 2_nd_ indicates that the first or second arginine is mutated to alanine. A summary of all the plasmids used is provided in Table S2. See also Figure S3.

The findings that mutations in *mdn1* substantially alter compound sensitivity indicate Rbins could directly target Mdn1, possibly by inhibiting its ATPase activity. As in other AAA+ proteins, ATP binding in Mdn1 is likely to be at the interface of two AAA domains and involves Walker A and B motifs from one AAA domain while the arginine finger motif extends from the following AAA domain (e.g., ATP binding at the AAA1 site depends on Walker A1, Walker B1, and the arginine finger R2 motifs) (Figures 2A and 2B) (Erzberger and Berger, 2006). Sequence alignment of Mdn1 homologs across different species, from yeast to human, showed that key motifs required for ATPase activity, including Walker A, Walker B, and Arginine Finger, are highly conserved for the AAA2-AAA5 sites (Kressler et al., 2012). AAA1 and AAA6 have conserved Walker A motifs but not Walker B motifs, suggesting that their ATP hydrolysis activity may not be required for Mdn1 function (Figure 2D). However, in contrast to dynein (Carter, 2013), the contributions of each ATP-binding site in Mdn1 have not been characterized.

**Figure S3:**
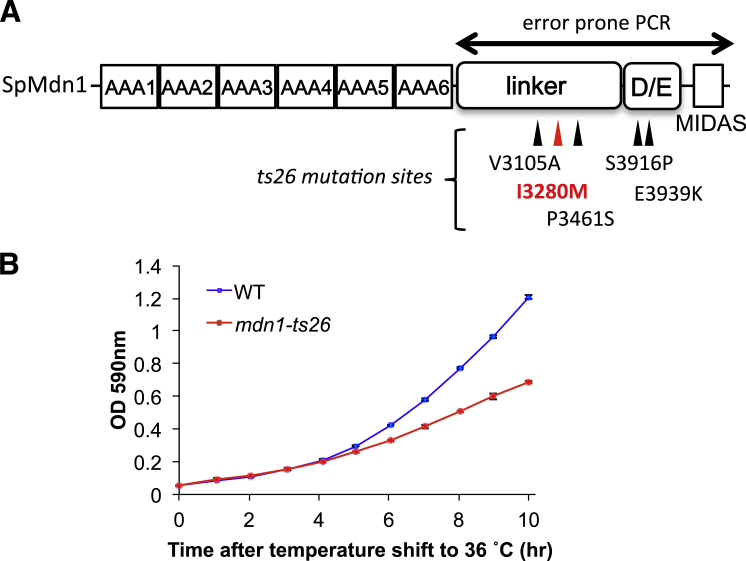
Construction of *mdn1-ts26* Mutant, Related to Figure 2 (A) The mutations in *mdn1-ts26* strain are shown. I3280M (red color) is the critical mutation that causes temperature sensitivity, since reversing only this mutation (I3280M was mutated to the original isoleucine) abolished temperature sensitivity (*mdn1ts26-IMI* in Figure 2C). (B) Growth curve of wild-type (WT) and *mdn1-ts26* cells at 36°C (mean±SDs, three independent experiments).

To address which ATP-binding pockets are essential for Mdn1’s functions, we established an “mdn1-ts complementation assay,” in which different Mdn1 mutants are overexpressed in an *mdn1* temperature-sensitive (ts) mutant strain and the growth at the restrictive temperature is measured. To develop this assay, we first generated an *mdn1* temperature-sensitive mutant strain (*mdn1-ts26,* Figures 2C, S3A, and S3B). We found that I3280M is the critical mutation that causes temperature sensitivity, since reversing only this mutation (I3280M was mutated to the original isoleucine) abolished temperature sensitivity (*mdn1ts26-IMI* in Figure 2C). We then generated the different ATPase mutants and expressed them in the *mdn1-ts26* strain (Figure 2D). Analysis of Walker A mutants showed that the A1 mutant suppressed temperature sensitivity of *mdn1-ts26* cells, similar to wild-type Mdn1, while others (A2–A6) did not (Figure 2E). Walker B mutants B2, B3, B4, and B5 failed to complement temperature sensitivity of the *mdn1-ts26* mutant (Figure 2E). Analyses of the arginine finger, which in the case of R2, R3, R4, and R6 can be two arginines and for R5 only one, showed that the double arginine to alanine R2 mutant suppressed temperature sensitivity of *mdn1-ts26* cells, while other mutants (R3–R6) did not (Figure 2E). Further analyses show that the second arginine residues at R3, R4, and R6 are critical for Mdn1’s functions (Figure 2F). As key residues in B_1_, B_6_ and R_1_ motifs are not conserved, they were not examined by mutagenesis. Together, these data suggest that the ATPase activity of AAA2, AAA3, AAA4, and AAA5 and ATP-binding ability of AAA6 are likely essential for Mdn1’s cellular functions.

### Characterization of Mdn1 ATPase Activity In Vitro

**Figure 3:**
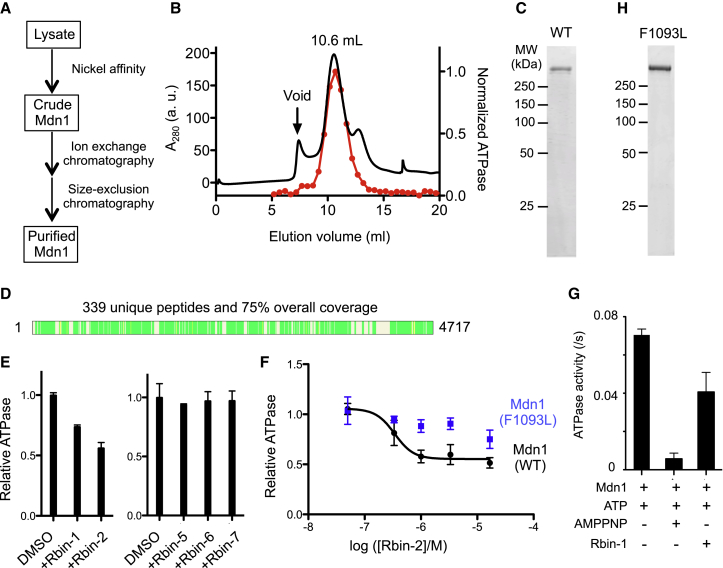
Rbins Inhibit Mdn1’s ATPase Activity In Vitro (A) Schematic for strategy used to purify recombinant full-length Mdn1. (B) The size-exclusion chromatography profile for Mdn1 (black trace). The activity of different fractions from this chromatography step was analyzed using a radioactive ATPase assay ([MgATP] = 0.1 mM). Activity was normalized to the most active fraction (red dots, each fraction; trace, interpolation). (C) SDS-PAGE analysis of purified full-length Mdn1 (WT) (Coomassie stain). (D) Peptides identified by mass spectrometry of the purified protein are indicated (green bars, schematic generated using Proteome Discoverer 1.4, Thermo Scientific). (E) Mdn1’s activity in the presence of Rbin analogs (1 μM) was tested using an NADH-coupled ATPase assay ([MgATP] = 1 mM). The relative activity is indicated (mean ± range, n = 2 independent experiments). (F) Dose-dependent inhibition of the steady-state ATPase activity of wild-type Mdn1 and Mdn1(F1093L) by Rbin-2 (mean ± SD, n = 4 independent experiments). An apparent EC_50_ was estimated for the inhibition of wild-type Mdn1 using a sigmoidal dose-response curve. Using similar equations, we were unable to properly fit the small decrease in activity of Mdn1(F1093L) across the concentration range tested. Unpaired t test (without Welch’s correction) of the measured activity for WT and Mdn1(F1093), at the highest three inhibitor concentrations tested, indicate that the difference in values are statistically significant (p = 0.0020 (at 16.7 μM), p = 0.0021 (at 3.33 μM), and p = 0.0019 (at 1.0 μM)). (G) The ATPase activity of Mdn1 in the presence Rbin-1 (1 μM) or AMP-PNP (2 mM) using the radioactive ATPase assay (MgATP = 0.1 mM). Error bars show SD (n = 6 independent experiments). (H) SDS-PAGE analysis of purified full-length Mdn1(F1093L) (Coomassie stain). See also Figure S4.

ATP hydrolysis by Midasin has not been demonstrated thus far. To analyze Mdn1’s ATPase activity and examine inhibition by Rbin-1, we needed to purify Mdn1 for in vitro biochemical assays. We tried different constructs and recombinant protein expression systems, including bacteria and yeast. A tandem-affinity protocol similar to the one used to purify recombinant Rea1 from budding yeast (Ulbrich et al., 2009) did not yield full-length fission yeast Mdn1. Finally, we employed the insect cell system to generate recombinant Mdn1. After significant optimization, we developed a multi-step strategy, which included nickel affinity, ion exchange, and size-exclusion chromatography (Figure 3A). In the final purification step, Mdn1 eluted from a size-exclusion column at a volume (10.6 mL, Void: 7.5 mL) consistent with its large size (mol wt ∼542 kDa for hexahistidine-tagged Mdn1) and extended shape based on analyses of Rea1 (Figures 3B and 3C) (Nissan et al., 2004, Ulbrich et al., 2009). A typical purification yielded ∼0.1 mg of recombinant Mdn1 per 3 L cell culture. Mass spectrometry confirmed that the single band resolved by SDS-PAGE was Mdn1, as we were able to identify 339 unique peptides that covered >70% of the Mdn1 sequence (Figure 3D). In particular, the identified peptides confirmed the presence of ∼80 amino acids at Mdn1’s C terminus. These data along with the N-terminal tag-based isolation indicate that we are able to purify full-length recombinant Mdn1.

To test recombinant Mdn1’s ATPase activity, we first analyzed the hydrolysis of radio-isotope labeled ATP. We found that the levels of ATPase activity closely matched the elution profile of Mdn1 from size-exclusion chromatography (Figure 3B, red trace). We next examined the steady-state ATPase activity of Mdn1 using an NADH-coupled enzyme assay. The specific activity of Mdn1 was ∼1.0 s^–1^ at 1 mM MgATP (Figure 3E). These data indicate that Mdn1 is an ATPase with specific activity comparable to that of other AAA+ proteins, such as VPS4 and dynein (Kon et al., 2004, Yang et al., 2015).

### Rbins Inhibit Recombinant Mdn1’s ATPase Activity In Vitro

We next examined inhibition of Mdn1’s steady-state ATPase activity by Rbin-1 and analogs. Two of the active analogs (Rbin-1 and Rbin-2) inhibited the ATPase activity by ∼40% at 1 μM (Figure 3E). Under similar conditions, analogs that were inactive in cell-based assays did not suppress ATPase activity (Figure 3E). As the amounts of Mdn1 were limiting, we focused on the dose-dependent inhibition of Mdn1 activity by Rbin-2, the most potent analog. While increasing concentrations of Rbin-2 resulted in greater inhibition, the dose-dependent inhibition saturated at ∼50%, consistent with inhibition of a subset of the ATPase domains in Mdn1. For the observed dose-response curve, we can estimate an apparent EC_50_ (∼0.3 μM, Figure 3F). For comparison, we find that almost complete inhibition of the ATPase activity (in a radioactive assay) can be achieved by a non-hydrolyzable ATP analog (AMPPNP, 2 mM), while Rbin-1 (1 μM) only inhibited activity by ∼40% (Figure 3G). The difference in potency of Rbins in in vitro ATPase and cell-based assays could be due to at least two reasons. First, full activity of Mdn1 may be needed for cell growth, and therefore partial inhibition (for example 15%) by Rbins could suppress growth. Second, the recombinant protein may not faithfully recapitulate the activity of the native protein, which may be post-translationally modified or could be in a complex with other proteins (e.g., Rsa4 in Rix1 particles) that may modulate activity or inhibition.

**Figure S4:**
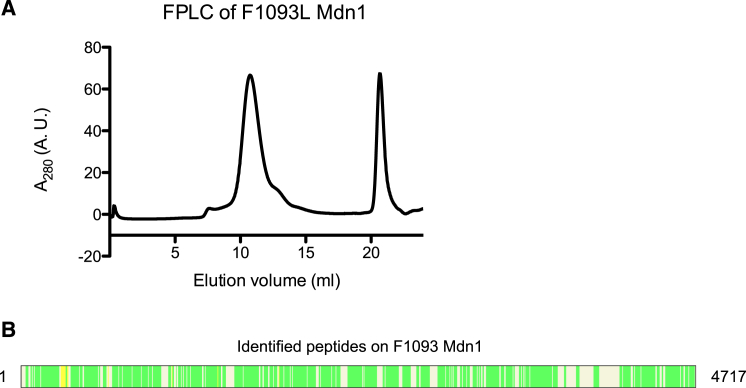
Purification of Recombinant Full-Length Mdn1 with the F1093L Point Mutation, Related to Figure 3 (A) Size-exclusion chromatography elution profile of recombinant F1093L Mdn1. (B) Mass spectrometry analysis of the single band from Figure 3H identified 256 peptides from F1093L Mdn1. (coverage: 71.5%). Green and yellow regions represent matched peptides with 1% and 5% false discovery rate, respectively (figure generated using Proteome Discoverer 1.4, Thermo Scientific).

To more firmly establish that Mdn1 is Rbins’ direct target, we generated full-length recombinant Mdn1 containing a mutation (F1093L) that confers resistance to the Rbins in cell-based assays (hereafter, Mdn1-F1093L, Figures 3H and S4). We find that Mdn1-F1093L is an active enzyme whose ATPase activity (1.5 ± 0.2 ATP s^–1^) in the steady-state assay is comparable to that of wild-type Mdn1. Importantly, Rbin-2 does not strongly suppress the ATPase activity of the Mdn1-F1093L (Figure 3F). Together, these data establish what we refer to as “gold standard” proof that the physiological target of Rbins is Mdn1, as the same single mutation can suppress inhibition by Rbins in both cell-based and in vitro biochemical activity assays (Wacker et al., 2012).

### Rbin-1 Allows Inhibition of Mdn1-Dependent Pre-60S Ribosome Biogenesis in Fission Yeast Cells on Fast Timescales

**Figure 4:**
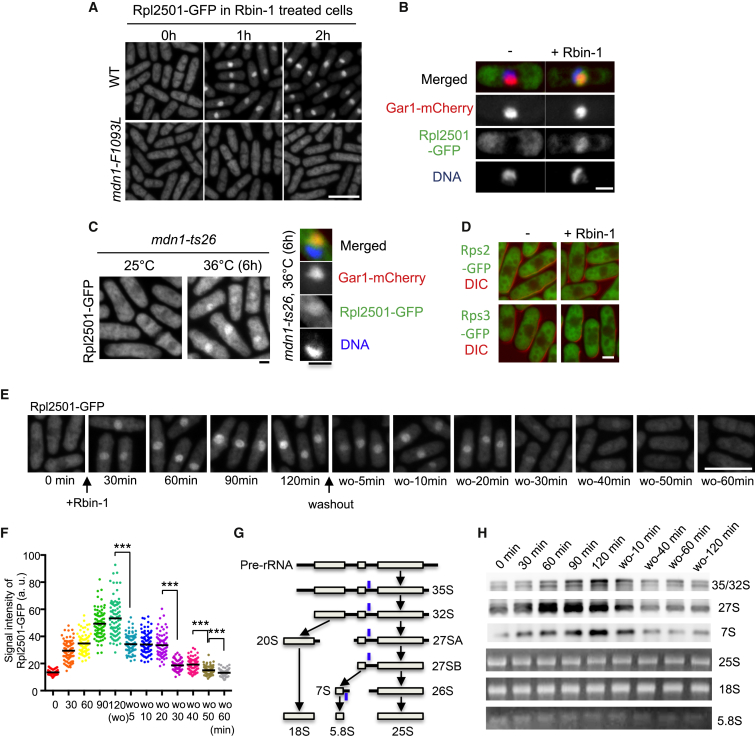
Chemical and Genetic Perturbations Indicate the Midasin Is Required for Nuclear Export of Pre-60S Particles and Pre-rRNA Processing in Fission Yeast (A) Rpl2501-GFP signals in wild-type (WT) and *mdn1-F1093L* cells with or without Rbin-1 treatment (1 μM) were examined. Scale bar, 10 μm. (B) Rpl2501-GFP signals (green) were compared with nucleolar marker Gar1-mCherry (red) and DNA (blue). Scale bar, 2 μm. (C) Rpl2501-GFP signals in *mdn1-ts26* cells at 25°C or 36°C for 6 hr were examined. Also shown are images of Rpl2501-GFP (green), Gar1-mCherry (red), and DNA stained with DAPI (blue). Scale bar, 2 μm. (D) The localization of markers for the 40S pre-ribosome, Rps2-GFP and Rps3-GFP (green, overlaid on the corresponding differential interference contrast [DIC] image, red), was examined in the presence or absence of Rbin-1 (1 μM). Scale bar, 2 μm. (E and F) Rpl2501-GFP localization was examined in asynchronous cells (0 min), 1 μM Rbin-1-treated cells (30–120 min), and in cells after washing out Rbin-1 (wo-5-60 min) at 29°C. Representative images are shown in (E). Scale bar, 10 μm. The signal intensity of Rpl2501-GFP in the nucleolus was measured in (F). Average is indicated by black bar (n = 80 cells). Asterisks indicate unpaired two-tailed Student’s t test significance value: ^∗∗∗^p < 0.001. (G) Schematic for pre-rRNA processing. The position of the northern blot probe, which can detect 35S/32S, 27S (27SA and 27SB), and 7S, is marked (blue line). (H) Northern blot (for 35/32S, 27S, and 7S) and GelRed staining (for 25S, 18S, and 5.8S) of total RNA. See also Figure S5.

**Figure S5:**
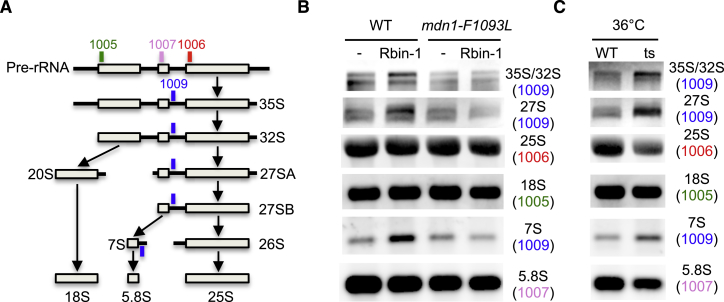
Analysis of Mdn1 Function Using Rbin-1 and a Temperature-Sensitive *mdn1-ts26* Mutant, Related to Figure 4 (A) Schematic for pre-rRNA processing. Positions of probes for Northern blot analyses are shown. (B) Exponentially growing culture of wild-type (WT) and the *mdn1-F1093L* cells were treated with or without Rbin-1 (1 μM) for 45 min at 32°C. Total RNA was isolated and analyzed by Northern blot. Detected rRNAs and the probes used for northern blots (1005–1007 and 1009) are indicated. (C) Exponentially growing culture of wild-type (WT) and *mdn1-ts26* (ts) cells were incubated for 6 hr at 36°C. Total RNA was isolated and analyzed by Northern blot. Detected rRNAs are indicated.

Guided by studies of Rea1 in *S. cerevisiae*, we examined Mdn1 function in *S. pombe* by taking advantage of Rbin-1 and genetics. We first examined pre-rRNA processing and the nuclear export of the pre-60S subunit. The defects in pre-rRNA processing were observed in Rbin-1-treated cells and in a *mdn1* temperature-sensitive mutant strain (Figure S5), as observed in budding yeast lacking Rea1 function (Galani et al., 2004). Importantly, these defects were not observed in Rbin-1-treated *mdn1-F1093L* mutant strains (Figure S5), indicating that the phenotype in Rbin-1-treated cells depends on Mdn1 inhibition. We also found that nuclear export of the pre-60S subunit, which can be tracked using Rpl2501-GFP, a homolog of budding yeast Rpl25 (Hurt et al., 1999), was blocked in Rbin-1-treated cells (Figures 4A and 4B). Importantly, this defect was not observed in Rbin-1-treated *mdn1-F1093L* mutant cells (Figure 4A). This defect was also observed at the restrictive temperature in a *mdn1* temperature-sensitive mutant strain (Figure 4C). In contrast, the nuclear export of the pre-40S subunits that were tracked using two different reporters, Rps2-GFP and Rps3-GFP (Galani et al., 2004), was not blocked in Rbin-1-treated cells (Figure 4D), indicating that Mdn1 has specific roles in pre-60S ribosome assembly. Further, by comparing Rpl2501-GFP with Gar1-mCherry (nucleolus marker) and DAPI (DNA marker), we found that pre-60S particles accumulated in the nucleolus upon Mdn1 inhibition (Figures 4B and 4C).

To examine the kinetics of ribosome assembly inhibition by Rbin-1, we examined Rpl2501-GFP levels at specific time points after inhibitor addition or washout. Upon Rbin-1 treatment the Rpl2501-GFP signal accumulated in the nucleolus within 30 min and reached a maximum level in 90–120 min (Figures 4E and 4F). Relief from Rbin-1 treatment revealed stepwise reductions in the nucleolar signal, with Rpl2501-GFP levels reducing substantially (∼47%) within 5 min of washout and then another ∼37% between 20 and 30 min and ∼15% between 40 and 60 min (Figures 4E and 4F). We also examined time-dependent defects in the processing of rRNA intermediates in Rbin-1-treated cells. The pre-rRNAs, such as 35S, 27S, and 7S pre-rRNA, accumulated only after 60 min of Rbin-1 treatment, reaching a maximum level 90–120 min after treatment (Figures 4G and 4H). The stepwise changes in Rpl2501-GFP levels in the nucleolus, observed upon RBin treatment or washout, also suggest that Midasin inhibition can lead to the accumulation of distinct intermediates of ribosome assembly.

### Analysis of Mdn1-Dependent Assembly of the Rix1 Particles in Fission Yeast

**Figure 5:**
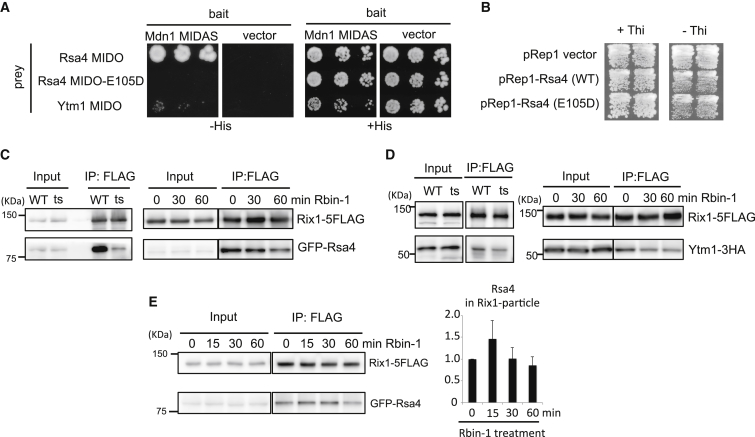
Analysis of Mdn1-Dependent Assembly of the Rix-1 Particles in Fission Yeast (A) Two-hybrid assay for the interaction between *S. pombe* Mdn1 MIDAS (4301–4717 amino acids [aa]) and *S. pombe* Rsa4 MIDO domain (1–144 aa, with or without E105D mutation) or *S. pombe* Ytm1 MIDO domain (1–93 aa). (B) Wild-type cells, in which wild-type (WT) or mutant (E105D) full-length Rsa4, or vector control (pRep1) were overexpressed from nmt1 promoter, were streaked on plates. Thiamine (Thi) addition represses transcription from nmt1 promoter. (C–E) Whole-cell extracts (Input) were prepared from wild-type (WT) and *mdn1-ts26* (ts) cells (incubated for 6 hr at 36°C) or wild-type cells treated with Rbin-1 (1 μM) for indicated times. Rix1-5FLAG was immunoprecipitated with anti-FLAG antibodies (IP: FLAG). The indicated proteins ([C] GFP-Rsa4, [D] Ytm1-3HA, [E] GFP-Rsa4) and Rix1-5FLAG were analyzed by SDS-PAGE and western blotting. The grouping of images from different parts of the same gel is indicated by dividing lines. The graph in (E) shows relative amount of Rsa4 in IP samples (mean ± range, n = 2). See also Figure S6.

**Figure S6:**
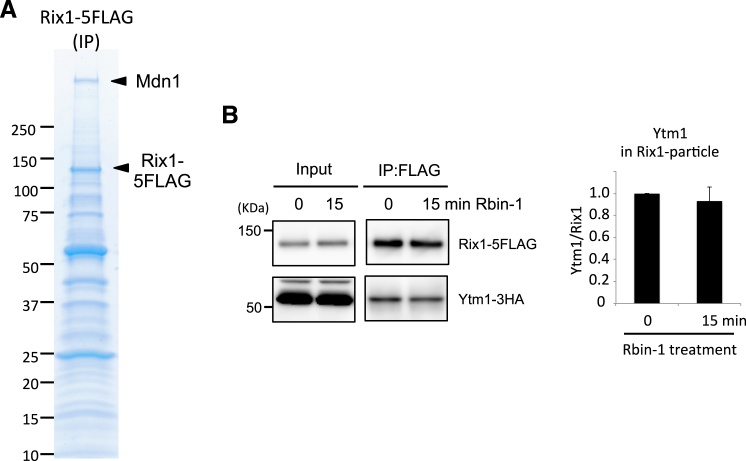
Additional Analysis of the Rix1 Particles, Related to Figure 5 (A) Whole cell extracts were prepared from cells expressing Rix1-5FLAG. Rix1-5FLAG was immunoprecipitated with anti-FLAG antibody. Co-immunoprecipitated proteins were analyzed by SDS-PAGE and Coomassie staining. Indicated protein bands were identified by mass spectrometry. (B) Co-immunoprecipitation of Rix1-5FLAG and Ytm1-3HA. Whole cell extract (input) were prepared from cells treated with Rbin-1 (1 μM) for indicated time. Rix1-5FLAG was immunoprecipitated with anti-FLAG antibodies (IP: FLAG). The indicated proteins were analyzed by SDS-PAGE and western blotted using anti-FLAG and anti-HA antibodies. Representative images are shown. The grouping of images from different parts of the same gel is indicated by dividing lines. The graphs show relative amount of Ytm1 in IP samples. (Error bars represent SD (n = 3)).

We next examined the roles of fission yeast Mdn1 in processing a well-characterized ribosome assembly intermediate, the Rix1 particle (Bassler et al., 2010, Ulbrich et al., 2009). Affinity purification of Rix1-5FLAG showed that Mdn1 is associated with the Rix1 particle (Figure S6A). In addition, similar to observations in budding yeast (Ulbrich et al., 2009), we find that fission yeast Mdn1 interacts with Rsa4 through conserved MIDAS-MIDO domains in a two-hybrid assay (Figure 5A). In comparison, similar analysis suggests that the interaction of fission yeast Ytm1 with Mdn1 is weaker than the Mdn1-Rsa4 interaction (Figure 5A).

In budding yeast, a point mutation of Rsa4 (E114D) that abolishes Rea1-Rsa4 interaction shows dominant-negative phenotypes (Ulbrich et al., 2009). In contrast, we found that overexpression of fission yeast Rsa4 with a point mutation (E105D), which abolishes Mdn1-Rsa4 interaction, did not show dominant-negative phenotypes (Figure 5B). Our data suggest that Mdn1-Rsa4 interaction is conserved between two yeasts but is likely not essential for fission yeast ribosome biogenesis.

Studies in *S. cerevisiae* predict that Rsa4 should be enriched in the Rix1 particle upon Mdn1 inhibition, as Mdn1 activity is required for the removal of Rsa4 from this pre-60S intermediate (Ulbrich et al., 2009). However, unexpectedly, we find that the amount of Rsa4 in Rix1 particles decreased at the restrictive temperature in our fission yeast *mdn1-*ts strain (Figure 5C). In addition, we find that Rsa4 levels were reduced in the Rix1 particles in fission yeast cells treated with Rbin-1 (Figure 5C). Similarly, Rix1-bound Ytm1 also decreased in *mdn1*-ts and Rbin-1-treated cells (Figure 5D). In a time-course experiment, we were able to detect 15 min after inhibitor treatment a slight increase in Rsa4 levels in the Rix1 particles (Figure 5E), consistent with a role for Mdn1 in removing Rsa4 from this pre-60S assembly intermediate. In comparison, Ytm1 levels in the Rix1 particles were largely unchanged at this time point (15 min, Figure S6B). However, at later time points the levels of Ytm1 and Rsa4 decreased (Figures 5C–5E), consistent with findings using the *mdn1-*ts strain. Together, these data support a role for Mdn1 in proper Rix1-particle assembly, and the time-dependent changes following Rbin treatments are consistent with Mdn1 contributing to more than one 60S-subunit processing step.

### Mdn1 Is Required for the Assembly of the Nucleolar Nsa1 Particles

**Figure 6:**
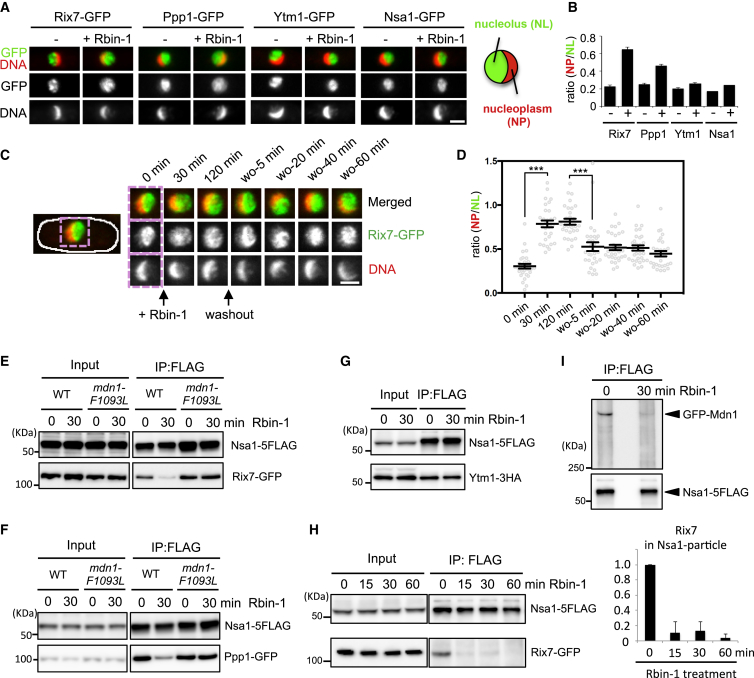
Mdn1 Is Required for the Assembly of the Nucleolar Nsa1 Particles (A and B) Rix7-GFP, Ppp1-GFP, Ytm1-GFP, and Nsa1-GFP signals in wild-type cells with or without Rbin-1 (1 μM, 1 hr at 25°C) treatment were examined. Representative images are shown in (A). Scale bar, 2 μm. The intensity of GFP signals in the nucleolus (NL) divided by that in nucleoplasm (NP) is shown in (B). Mean ± SEM, n = 30 cells. (C and D) Rix7-GFP signals were examined in asynchronous cells (0 min), 1 μM Rbin-1-treated cells (30–120 min), and cells after washing out Rbin-1 (wo-5-60 min) at 29°C. Representative images are shown in (C). Scale bars, 2 μm. The intensity of the Rix7-GFP signal in the nucleolus (NL) divided by that in the nucleoplasm (NP) is shown in (D). Average is indicated by black bar (mean ± SEM, n = 30 cells). Asterisks indicate unpaired two-tailed Student’s t test significance value: ^∗∗∗^p < 0.001. (E–I) Whole-cell extracts (Input) were prepared from wild-type or *mdn1-F1093L* cells treated with Rbin-1 (1 μM) for indicated time. Nsa1-5FLAG was immunoprecipitated with anti-FLAG antibodies (IP: FLAG). The indicated proteins ([E] Rix7-GFP, [F] Ppp1-GFP, [G] Ytm1-3HA, [H] Rix7-GFP, and [I] GFP-Mdn1) and Nsa1-5FLAG were analyzed by SDS-PAGE and western blotting. The grouping of images from different parts of the same gel is indicated by dividing lines. The graph in (H) shows relative amount of Rix7 in IP samples (mean ± range, n = 2). See also Figure S7.

We considered the possibility that Mdn1 contributes to nucleolar ribosome assembly steps that precede the formation of the nucleoplasmic Rix1 particle. One of the characterized nucleolar pre-60S particle in budding yeast is the Nsa1 particle that contains several assembly factors, such as Rix7, Ppp1, and Ytm1 (Kressler et al., 2012). We first examined the localization of Nsa1 particle-associated proteins in untreated and Rbin-1-treated cells. Rix7, Ppp1, Ytm1, and Nsa1 predominantly localized in the nucleolus of growing fission yeast cells (Figures 6A and 6B). Intriguingly, we find that Rix7 and Ppp1, but not Ytm1 and Nsa1, mislocalized in Rbin-1-treated cells (Figures 6A and 6B). We further examined the kinetics of Rix7 localization upon Mdn1 inhibition and activation and found that Rbin-1 treatment for 30 min resulted in maximum observed change in the nucleoplasmic Rix7 signal, and, within 5 min after washing out Rbin-1, Rix7 redistributed (Figures 6C and 6D). The rapid reduction of the fluorescence signal illustrates the fast timescales of Rbin-1 action. In addition, these data suggest a role for Mdn1 in ribosome assembly steps that precede the formation of the Rix1 particle.

**Figure 7:**
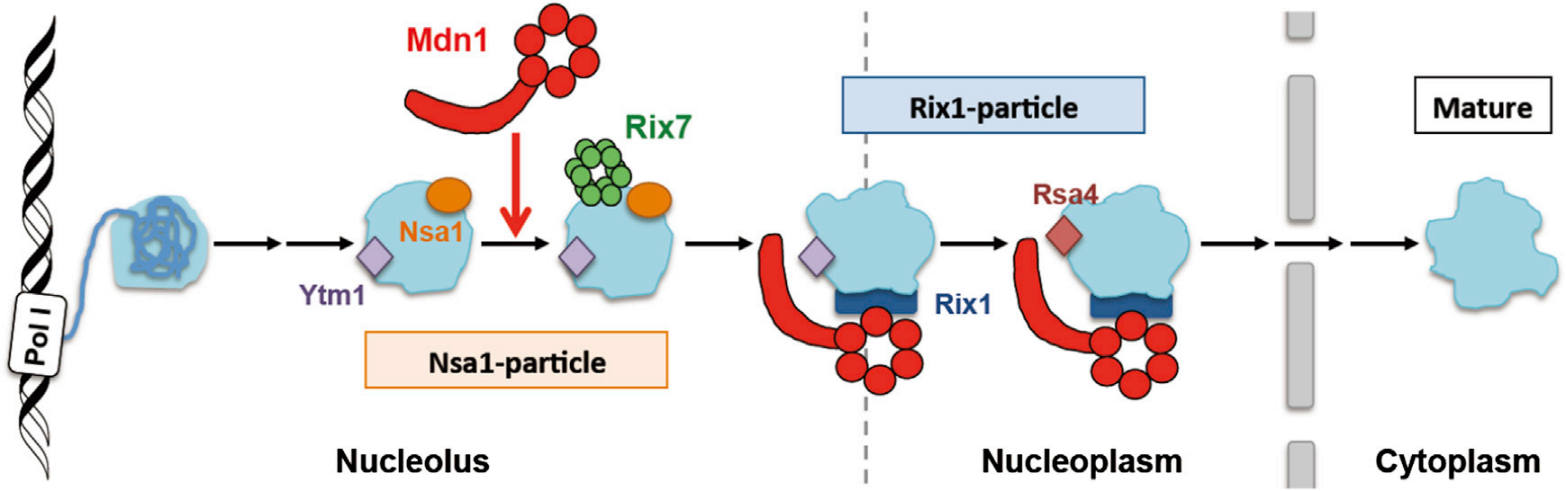
Model for Mdn1’s Functions during the Assembly of the 60S Subunit of the Ribosome In the nucleolus, Mdn1 binds to the pre-60S particle containing Nsa1 and Ytm1 (Nsa1 particle) and helps recruit Rix7, another AAA+ protein. Mdn1-dependent removal of Ytm1 allows pre-60S particles to exit the nucleolus, and Mdn1-dependent removal of Rsa4 from the Rix1 particle allows pre-60S particles to leave the nucleoplasm.

**Figure S7:**
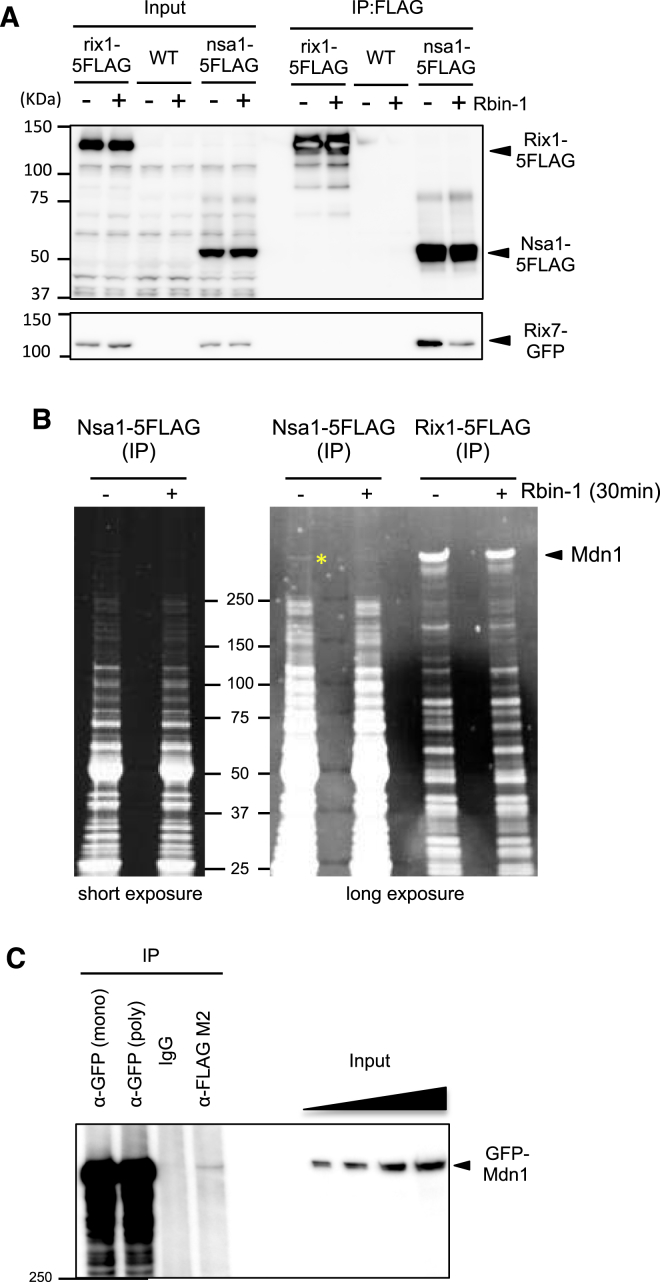
Additional Analysis of the Nsa1 Particle, Related to Figure 6 (A) Whole cell extract (Input) were prepared from cells treated with (+) or without (-) Rbin-1 (1 μM) for 30 min at 29°C. Rix1-5FLAG or Nsa1-5FLAG was immunoprecipitated with anti-FLAG antibodies (IP: FLAG). The indicated proteins were analyzed by SDS-PAGE and western blotting using anti-FLAG and anti-GFP antibodies. (B) Nsa1-5FLAG or Rix1-5FLAG was immunoprecipitated with anti-FLAG antibody from cells treated with (+) or without (-) Rbin-1 (1 μM) for 30 min at 29°C. IP samples were analyzed by SDS-PAGE and ORIOLE staining. Mdn1 bands in the Rix1-5FLAG IP sample were identified by mass spectrometry. The band indicated by yellow asterisk is likely Mdn1 by molecular weight. Confirmation of this band by mass spectrometry failed, likely due to low protein levels. Rbin-1 treatment did not change overall composition of Rix1- and Nsa1-particles, while Mdn1 in the Nsa1-particle is likely reduced by Rbin-1 treatment. (C) Co-immunoprecipitation of GFP-Mdn1 and Nsa1-5FLAG. Whole cell extract (Input) were prepared from cells expressing GFP-Mdn1. Nsa1-5FLAG or GFP-Mdn1 was immunoprecipitated with anti-FLAG, monoclonal anti-GFP (mono), or polyclonal antibodies anti-GFP (poly) antibodies. GFP-Mdn1 was analyzed by SDS-PAGE and western blotted using anti-GFP antibody.

We next purified and analyzed the Nsa1 particles in Rbin-1-treated cells and compared them to untreated controls. The amount of Rix7 and Ppp1 in the Nsa1 particles was significantly reduced by Rbin-1 treatment, while the amount of Ytm1 in the Nsa1 particle remained relatively constant (Figures 6E–6G and S7A). Importantly, a Rbin-1-resistant *mdn1-F1093L* mutation suppressed these changes (Figures 6E and 6F), indicating that the phenotypes due to Rbin-1 treatment depended on Mdn1 inhibition. Time-course experiments show that Rix7 reduction was detected within 15 min after Rbin-1 treatment (Figure 6H). Consistent with a role for Mdn1 in Nsa1-particle assembly, we find that Mdn1 is present in this particle in fission yeast (Figure 6I; Figures S7B and S7C). Further, Rbin-1 treatment substantially reduced the levels of Mdn1 in the Nsa1 particle (Figure 6I). The amount of Mdn1 co-purified with Nsa1 was less than that pulled down with Rix1 (Figure S7B), suggesting a more transient or sub-stoichiometric association. Taken together, these data suggest that Mdn1 activity is required for maintaining Mdn1 itself in the nucleolar Nsa1 particle, where Mdn1 may help recruit ribosome assembly factors, such as Rix7 and Ppp1 (Figure 7).

## Discussion

In this study, we report the discovery of ribozinoindoles, potent and selective chemical inhibitors for Midasin, an essential AAA+ protein required for eukaryotic ribosome biogenesis. Matched inhibitor-sensitive and inhibitor-resistant cells allow systematic analyses of dose-dependent target-specific effects of the chemical probe, addressing a major potential limitation of the use of chemical inhibitors to examine cellular mechanisms. Further, the fast timescale of Mdn1 inactivation and activation by Rbins is well suited to dissect Mdn1's role in coordinating the spatial and temporal dynamics of ribosome biogenesis.

Eukaryotic ribosome biogenesis involves complex multi-step RNA processing, the recruitment of ribosomal proteins and dynamic associations of multiple non-ribosomal proteins (Thomson et al., 2013). Briefly, two rRNA precursors, the 35S and the pre-5S, are transcribed in the nucleolus. The 35S RNA is processed to generate the 27S RNA that is subsequently cleaved to generate 25.5S and 7S RNAs. These RNAs contain sequences corresponding to the 25S and 5.8S rRNAs present in the mature 60S subunit. Findings from studies in budding yeast suggest that Mdn1/Rea1 is involved in two steps of ribosome biogenesis. First, Mdn1/Rea1 removes Ytm1 from pre-60S particles likely before the cleavage of an internal transcribed spacer (ITS2) from the 27S RNA. This step is needed for the exit of pre-60S particles from the nucleolus to the nucleoplasm (Bassler et al., 2010). Second, Mdn1/Rea1 dissociates Rsa4 from the pre-60S particles in the nucleoplasm, before or during the processing of 7S pre-rRNA (Ulbrich et al., 2009). This Mdn1/Rea1 function is needed for the pre-60S particles to be exported from the nucleoplasm to the cytoplasm (Bassler et al., 2010, Ulbrich et al., 2009) (Figure 7). Similar to what has been found in budding yeast for Rea1, *S. pombe* Mdn1 can interact with Rsa4 and Ytm1 through conserved MIDAS-MIDO domains. In addition, consistent with this model, we find that both 27S and 7S pre-rRNA processing steps and export of pre-60S particles from the nucleolus are blocked in Rbin-1-treated fission yeast cells. Finally, the rapid, though transient, increase of Rsa4 in the Rix1 particle upon Rbin-1 treatment supports the proposed function of Mdn1/Rea1 at this step of ribosome biogenesis. Together, these data indicate that many aspects of Mdn1/Rea1-dependent ribosome biogenesis are likely to be conserved across fungi.

Our studies in fission yeast also suggest an additional role for Mdn1 during ribosome biogenesis. We propose that Mdn1 contributes to the assembly of the Nsa1 particles in the nucleolus based on the following three lines of evidence. First, Rix7 and Ppp1 have reduced association with the Nsa1 particles upon Mdn1 inhibition. Second, we observe activity-dependent association of Mdn1 with the Nsa1 particles. Third, upon Rbin-1 treatment, key ribosome assembly factors that bind the Nsa1 particles, such as Rix7 and Ppp1, disperse in the nucleus rather than accumulate in the nucleolus. Analysis of Rpl2501-GFP signals in Rbin-1-treated cells suggests that pre-60S particles are mainly accumulated in the nucleolus upon loss of Mdn1 function (Figure 4B). Therefore, the mislocalization of Rix7 and Ppp1 is likely due to the loss of Mdn1-dependent association of these proteins with the nucleolar pre-60S particles. Studies in budding yeast indicate that Ytm1 is enriched in the Nsa1 particles among distinct pre-60S particles in the nucleus (Bassler et al., 2010, Ulbrich et al., 2009). As Ytm1 levels in the Nsa1 particle are not changed upon Mdn1 inhibition (Figure 6G), we propose that Mdn1 has a specific function in pre-60S processing in the nucleolus, after Ytm1 associates with the Nsa1 particle and before Ytm1 is removed from the particle by Mdn1 (Figure 7). Additional work is needed to identify the specific nucleolar pre-60S precursor that contains Ytm1, Rix7, Ppp1, and Mdn1.

Importantly, the timescale of Midasin inactivation and activation by Rbin-1 (5–15 min) is significantly faster than what we can achieve using genetic approaches (∼3–6 hr using our temperature-sensitive allele). In particular, the temporal control over target function that we can achieve with these chemical probes is evident from studies of Rpl2501 and Rix7 localization, which are altered in cells within 5 min of inhibitor washout (Figures 4 and 6). Analyses of the timescales over which different phenotypes accumulate following chemical inhibitor treatments can guide interpretations and help develop models for target protein function in a dynamic, ordered, multi-step cellular process, such as eukaryotic ribosome biogenesis. For example, immediately after Rbin-1 treatment the levels of Rsa4 in Rix1 particles increase (Figure 5E), consistent with the proposed function of Mdn1 in the assembly of these pre-60S particles (Ulbrich et al., 2009). However, after prolonged chemical inhibition, or genetic suppression of Mdn1 in fission yeast, Rsa4 levels in the Rix1 particles drop (Figures 5C and 5E), observations that do not support the proposed model. Importantly, we find reduction in Rsa4 levels in the Rix1 particles after Rbin-1 treatment requires more time than the reduction of Rix7 in the Nsa1 particles. Therefore, it is likely that inhibition of the “upstream” Nsa1-particle assembly leads to defects that accumulate and alter the flux of pre-60S precursors and indirectly, over extended time, impact the levels of proteins, such as Rsa4, associated with Rix1. Developing a precise order of assembly and disassembly for proteins during ribosome biogenesis will require additional biochemical characterization of ribosome assembly intermediates. There are several technical issues, such as the compositional heterogeneity of particles isolated using single tag-based purifications, which need to be addressed. For example, it remains unclear how many distinct sub-particles containing Rix1 exist in the nucleolus and nucleoplasm. It is likely that Rbins, which allow acute and reversible inhibition of Mdn1, will help dissect these fast assembly dynamics.

Midasin and the closely related motor protein dynein are the only known members of the AAA+ protein family that have all six AAA domains on a single polypeptide, a feature that likely allows functional specialization of each of the multiple AAA domains (Carter, 2013). In the case of dynein, which has been extensively studied over the last couple of decades, two of its ATPase sites contribute to the overall ATP hydrolysis and directional transport activity (Carter, 2013). As Rbin resistance and sensitivity conferring mutations cluster proximal to one ATP binding site in Mdn1, we favor the possibility that Rbins act through inhibiting the activity of the ATPase site formed by AAA3 and AAA4 domains. Consistent with this proposal, we find that mutations of Walker A, Walker B, or Arginine-finger residues at this ATPase site suppress cell growth. If Rbins target only a single ATPase site in Mdn1, our data suggest that this ATPase site contributes to half of the total activity and the other sites contribute to the remainder. Additional structural and biochemical studies will be needed to test this hypothesis.

Our discovery of a specific and potent chemical inhibitor of Midasin, along with the findings that dynein and p97/VCP can be selectively inhibited by cell-permeable low-molecular-weight compounds (Anderson et al., 2015, Chou et al., 2011, Firestone et al., 2012), indicates that drug-like inhibitors can be developed for AAA+ proteins. These discoveries lay the foundation for developing pharmacophore models for rationally designing chemical inhibitors for AAA+ proteins for which we lack chemical probes, such as proteins involved in DNA replication, mitochondrial function, or cytoskeleton organization.

## STAR★Methods

### Key Resources Table

**Table:** 

REAGENT or RESOURCE	SOURCE	IDENTIFIER
**Antibodies**		

Mouse monoclonal anti-GFP	Roche	Cat# 11814460001
Mouse M2 monoclonal anti-FLAG	Sigma	Cat# F3165
Mouse 12CA5 monoclonal anti-HA	abcam	Cat# ab16918

**Chemicals, Peptides, and Recombinant Proteins**		

Cycloheximide	Sigma	Cat# C7698
Rbin-1 and analogs (see General Synthesis Information)	synthesized	N/A
Roche Complete, EDTA-free Protease Inhibitor	Roche	Cat# 11873580001

**Critical Commercial Assays**		

DIG Northern Starter Kit	Roche	Cat# 12039672910

**Experimental Models: Organisms/Strains**		

See Table S1 for *S. pombe* strains	This paper	N/A
*S. cerevisiae*: AH109 strain	Masayuki Yamamoto lab (NIBB)	N/A

**Recombinant DNA**		

pDUAL-YFH1c-mdn1	Minoru Yoshida lab (RIKEN)	N/A

**Software and Algorithms**		

GraphPad Prism	GraphPad Software	N/A

### Contact for Reagent and Resource Sharing

Further information and requests for reagents may be directed to, and will be fulfilled by Tarun M. Kapoor (kapoor@mail.rockefeller.edu) at the Rockefeller University.

### Experimental Model and Subject Details

#### S. pombe

All strains used are listed in Table S1. Standard growth conditions and methods were used (Moreno et al., 1991). Most experiments were performed in yeast extract (YE) medium containing adenine, leucine, uridine, and histidine. All plasmids used are listed in Table S2. To overexpress Mdn1 by the nmt1 promoter, pRep1-based plasmids were transformed into host strain, and transformants were incubated in minimal medium (EMM) without thiamine and leucine for over 15 hr. PCR-based gene targeting (Bähler et al., 1998) was used to construct gene deleted and fluorescent protein-tagged strains with selection marker gene cassettes. The MDR-sup strain is an engineered drug-sensitive fission yeast, in which 7 MDR genes were inactivated (Aoi et al., 2014, Kawashima et al., 2012). For generating *mdn1-ts26 < < kanr* strain, PCR-based random mutagenesis was performed. The 10 kb DNA fragment containing *mdn1* Linker, D/E rich, and MIDAS domains, along with *mdn1* 3′-UTR and *kanMX* marker, was amplified by LA Taq (Takara). The PCR reaction was performed for 45 cycles to introduce mutations. The amplified DNA fragment was used for transformation of *h*^*90*^ *leu1 ade6-M216* strain, and colonies that formed at 25°C but not at 36°C were isolated. For generating *mdn1-ts26-IMI < < kan*, the *mdn1-ts26* strain was transformed using the 1 kb DNA fragment containing the wild-type I3280 codon sequence, followed by isolation of survivors at 34°C. For generating *kan < < mdn1-L1113F* strain, DNA fragment containing *kan < < mdn1-L1113F* was transformed into *h*^*+*^ *ade6-M210 leu1 ura4-D18* strain, and G418-resistant colonies were selected. The *mdn1-L1113F* mutation was confirmed by sequencing. To construct rpl2501-GFP strain, DNA fragment containing *leu1N(the first 150bp)-P*_*adh1*_*-rpl2501-GFP-T*_*nmt1*_*-ura4*^*+*^*-leu1C (the last 150bp)* was transformed into *h- ura4-D18* strain, and Ura^+^ Leu^−^ colonies were selected. To construct the *GFP-mdn1* strain, the DNA fragment carrying *ura4+-Pnmt1-GFP-linker* was integrated at the downstream of *mdn1* promoter (*P*_*mdn1*_) in the genome by homologous recombination, followed by deletion of the *ura4+-P*_*nmt1*_ region using the DNA fragment containing *P*_*mdn1*_*-GFP* and isolation of FOA-resistant transformants.

### Method Details

#### Chemical synthetic lethal screen

SAK84 and SAK236 strains (Table S1) were used for the chemical genetic screen. The chemical libraries that were used for screen were described previously (Takemoto et al., 2016). The MDR-sup strains are better suited for chemical screens as lower concentrations of compounds can be used than what is needed when using wild-type strains. Logarithmically growing cells (OD = 0.5) were diluted 50-fold, mixed with compounds (2 μM) and YE medium (total volume: 50 μl per well), and incubated for 15 hr at 29°C. Multidrop Combi (Thermo Scientific) was used to dispense the cells into wells of the 384-plate (Greiner clear flat bottom PS plate). The growth was measured using a microtiter plate reader (Perkin-Elmer EnVision, 590 nm filter). For calculation of growth ratios, OD values of each well were divided by that of control well incubated with DMSO. From 10,353 compounds, we identified 6 compounds, including Rbin-1, that were more toxic to the *cut2-364* strain than the wild-type strain.

#### Isolation and construction of Rbin-1-resistant mdn1 mutants

For *mdn1* mutagenesis, the SAK72 (Table S1) was transformed with *mdn1* fragment (1-4378bp) amplified by error-prone PCR, and spread onto plates containing 5 μM Rbin-1. 11 resistant clones were isolated. Sequencing of these clones revealed that all resistant clones had a point mutation (D1123E: 9 clones, and E1187K: 2 clones) in the *mdn1* gene. For unbiased mutagenesis, the SAK72 strain treated with 1-methyl-3-nitro-1-nitrosoguanidine (NTG) in TM buffer (50 mM Tris, 50 mM Maleic acid, 7.5 mM (NH_4_)_2_SO_4_, 0.4 mM MgSO_4_·7H_2_O [pH 6.0]) for 30 min, and incubated in YE medium for > 3 hr, spread onto plates containing 5 μM Rbin-1. 13 resistant clones were isolated. Sequencing of these clones revealed that all resistant clones had a point mutation (F1093L: 6 clones, F1093S: 2 clones, F1097S: 2clones, L1335S: 2 clones, and E1044V: 1 clone) in the *mdn1* gene. To re-construct these *mdn1* point mutants, a DNA fragment containing a *mdn1* point mutation, which was amplified by PCR from the genome of each Rbin-1-resistant mutant, was transformed into the SAK72 strain. Homologous recombination between *mdn1* mutant fragments and the *mdn1*^*+*^ gene produced *mdn1* mutant strain. Rbin-1-resistant clones were selected on 5 μM Rbin-1 containing plates. The replacement of *mdn1*^*+*^ gene by *mdn1* mutant was confirmed by sequencing. All resistant clones in MDR-sup background were crossed with the wild-type strain to construct *mdn1* point mutants in the wild-type background.

#### SAR analysis of Rbin analogs using growth assay of pombe

*S. pombe* strains were grown to exponential phase and diluted to OD_600_ ∼0.01. 1 μl DMSO or DMSO solution of Rbins (1000x) were mixed with 1 ml diluted cell suspensions. The cells were placed in shaker (220 rpm) for 18 hr at 29°C till OD_600_ of DMSO control reached ∼1. Relative growth was calculated by dividing the measured OD at a specific concentration by the OD for the DMSO control. Half maximum growth inhibition (GI_50_) was determined by fitting relative growth to a four-parameter sigmoidal dose-response curve in PRISM.

#### Construction of full-length *S. pombe* mdn1

For the wild-type *mdn1* gene, DNA fragments were PCR-amplified from pDUAL-YFH1c-mdn1 (gift from Minoru Yoshida) and *S. pombe* genomic DNA. After ligation of DNA fragments, the full-length *mdn1* gene was cloned into pREP1NTAPΔNdeI vector or pFastBac HTC vector. For the *mdn1* ATP site mutants (e. g. Walker A, Walker B, and R finger), mutations were introduced into N (1-3355 bp) or M (3350-8528 bp) fragment of the *mdn1* gene using PrimeSTAR Mutagenesis Basal Kit (TAKARA), followed by ligation of N, M, and C (8523-14154 bp) fragments. The full-length *mdn1* mutant genes were cloned into pREP1NTAPΔNdeI vector or pFastBac HTC vector.

#### mdn1-ts complementation assay

The SAK2189 (*mdn1-ts26*) strain was transformed with wild-type or mutant pREP1NTAPΔNdeI-*mdn1* plasmid, and grown on EMM–Leu plate containing 30 μM thiamine at 25°C. Colony PCR and DNA sequencing was carried out to confirm that transformants harbored correct plasmid DNA. 5 ml EMM–Leu pre-cultures containing 30 μM thiamine were incubated overnight at 25°C up to OD (590 nm) ∼0.5. Cells were washed with DDW to remove thiamine, followed by resuspension in thiamine-free EMM–Leu medium at OD (590 nm) = 0.01. After incubation of 2 ml culture for 24 hr at 36°C, OD (590 nm) values were measured.

#### Purification of recombinant *Schizosaccharomyces pombe* Mdn1

All biochemical reagents were obtained from SIGMA-ALDRICH unless specified otherwise. The cDNA encoding fission yeast Mdn1 was cloned into pFastBac HTC vector (Invitrogen). We used the Bac-to-Bac system (Invitrogen) to generate recombinant baculovirus. High Five cells (Life Technologies) were grown in Sf-900 II SFM (Life Technologies 10902-096) with 1X Antibiotic-Antimyocotic (Life Technologies) to ∼2.5 million/mL and then infected (1:80 dilution of P2 virus). The cells were harvested 60 hr after infection. All of the following steps were done on ice or at 4°C. The cells were lysed by sonication in equal volume of lysis buffer (50 mM Tris, 400 mM NaCl, 20 mM imidazole, 1 mM MgCl_2_, 5 μM 2-mercaptoethanol, 200 μM ATP, 3 U/mL benzonase, 1X Roche complete protease inhibitor without EDTA, 10% glycerol [pH 7.5]). The homogenized lysate was then centrifuged at 55,000 rpm for 1 hr. The supernatant was incubated with Ni-NTA beads (QIAGEN) for 40 min. The beads were extensively washed using Washing buffer (50 mM Tris, 400 mM NaCl, 20 mM imidazole, 1 mM MgCl_2_, 5 μM 2-mercaptoethanol, 10% glycerol [pH 7.5]). The protein was then eluted by high imidazole buffer (20 mM Tris [pH 7.5], 120 mM NaCl, 300 mM imidazole, 1 mM MgCl_2_, 5 μM 2-mercaptoethanol, 200 μM ATP (A2383)). The eluted fraction was filtered and loaded onto a Mono Q column 5/50 GL (GE Healthcare Life Sciences). The protein was eluted around 400 mM NaCl and the fractions were collected and analyzed by SDS-PAGE. The relevant fractions were pooled and then concentrated using Amicon Ultra-4 Centrifugal Filter Units. The concentrated sample was then loaded on Superose 6, 10/300 GL (GE Healthcare Life Sciences) using FPLC SEC buffer (20 mM Tris (pH = 7.5), 150 mM NaCl, 1 mM MgCl_2_, 1mM EGTA and 5 μM 2-mercaptoethanol). Peak fractions were collected and ATPase assay was carried out directly with fresh protein. Protein concentration was determined using a Bradford assay.

#### Radioactive ATPase Assay

Radioactive γ-P32-ATP (PerkinElmer, BLU002Z250UC) was added to 600 μM MgATP (pH = 7.0) solutions at volume ratios of 1:1000-1:300, depending on the lifetime of the radioactive reagent. The total volume of each reaction was 12 μL, including 6 μl of protein from size exclusion chromatography fractions (final concentration ∼0-50 nM for different fractions, peak fractions were used for Rbin-1 and AMPPNP inhibition experiments in Figure 3E), 4 μl FPLC SEC buffer with 0.6 mM Na_2_SO_4_ and 2 μl MgATP (final concentration = 100 μM). The reactions were then incubated at room temperature for 30 or 60 min before quenching with 12 μl 0.2 M EDTA. 1 μl from each reaction mixture was spotted on to TLC PEI cellulose F plates (Millipore, 105579). The TLC buffer contained 0.15 M formic acid and 0.15 M lithium chloride. The TLC plates were then imaged using the Typhoon Scanner 9400 (GE Healthcare Life Sciences). ImageJ was used to calculate the densitometric ratio of the spots corresponding to radioactive free phosphate and ATP to determine the percent of ATP hydrolyzed.

#### NADH-coupled steady-state ATPase assay

For each steady-state ATPase reaction the final volume was 30 μL. The final concentration of protein was 50 nM. The FPLC SEC buffer was used to dilute the sample. NADH (N7410), phosphoenol pyruvic acid monopotassium salt (P7127), D-lactic dehyrogenase (L3888) and pyruvate kinase (ammonium sulfate suspension, P1506) were added to final concentration of 140 μM, 1.25 mM, 40 U/mL and 80 U/mL. 1 μl DMSO/compound solution was added and mixed. 3 μl 10 mM MgATP (pH = 7.0), was added to make the final total volume of 30 μl and final concentration of MgATP was 1 mM. Time course of fluorescence decrease due to NADH oxidation was measured using Synergy NEO Microplate Reader. The fluorescence values were plotted against time and fit by linear regression. The slopes of these lines were used to calculate the ATPase rate. The relative activities were obtained by dividing the measured activity by activity in DMSO control (no compound). The EC_50_ value for the inhibition of wild-type Mdn1 was determined by fitting the average values for relative activity at each inhibitor concentration (n = 4 independent measurements) to a four-parameter sigmoidal dose-response curve using PRISM.

#### Image acquisition

For all the microscopy experiments cells were fixed using methanol, stained with DAPI, imaged by a microscope (Axio Imager 2; Carl Zeiss) with a 63x objective (Plan-APOCHROMAT, 63x/1.4 Oil DIC; Carl Zeiss), and processed with Axio Vision 4.8 software (Carl Zeiss). A Z-stack of ∼2 μm thickness, with single planes spaced by 0.3 μm, was acquired and maximum intensity projections were generated. To compare signal intensities, all images were taken with the same exposure conditions and processed similarly.

#### Two hybrid analysis

The AH109 strain was co-transformed with pGBKT7-mdn1 MIDAS as the bait plasmid and pGADT7-rsa4 MIDO, pGADT7-rsa4 MIDO-E105D, or pGADT7-ytm1 MIDO as the prey plasmids. Representative transformants were spotted on SC–LeuTrp (+His) and SC–LeuTrpHis (–His) plates in 1:5 dilution series, and incubated for 2 days at 32°C.

#### Quantification of GFP signals in nucleoplasm and nucleolus

Interphase *S. pombe* cells have crescent-like shaped nucleoplasm (or DAPI-positive region) as shown in the cartoon (Figure 6A). Because of the different orientations of cells, some interphase cells show clear crescent-shape, but the others do not in projected single images. Therefore, for quantification, cells that showed clear crescent-shape DAPI signals were randomly chosen, and then GFP signals in nucleoplasm (DAPI-positive region) and/or nucleolus (DAPI-negative region) were quantified. The signal intensity of GFP signals was plotted in GraphPad Prism 6 to quantify nucleolar Rpl2501-GFP signals, and ratio of nucleoplasmic and nucleolar Rix7-GFP signals. The ratios of nucleoplasmic and nucleolar signals of Rix7-GFP, Ppp1-GFP, Ytm1-GFP, and Nsa1-GFP in Figure 6B were calculated.

#### RNA analysis

Total RNA was isolated from *S. pombe* cells using the hot phenol method followed by phenol-chloroform extraction, precipitation, and purification using QIAGEN RNeasy columns (Lyne et al., 2003). Northern blots were performed using the DIG Northern Starter Kit (Roche) according to the manufacturer’s protocol. Denatured total RNAs were separated in 1.3% agarose gel containing 1.85% formaldehyde, 20 mM MOPS, 5 mM sodium acetate, and 1 mM EDTA. To detect abundant rRNAs (25S, 18S, and 5.8S), gels were stained by GelRed (Biotium). For Northern blotting, gels were transferred to positively charged nylon membrane (Hybond-N, GE Healthcare) using Whatman TurboBlotter Transfer System (Fisher Scientific). After RNAs were fixed to the membrane by UV-crosslinking (120 mJ), the membrane was incubated with hybridization buffer (DIG Easy Hyb Granules with 0.1 mg/ml Poly (A) and 5 μg/ml Poly (dA)) for blocking at 40°C for 30 min, and then with hybridization buffer containing 21.7 pmol digoxigenin (DIG)-labeled probes generated using DIG Oligonucleotide Tailing Kit, 2nd generation (Roche) at 40°C for 6 hr. The primers for generating probes were 1005 (5′- CTTAGACATGCATGGCT −3′), 1006 (5′- GCGCTTATTGATATGCTT −3′), 1007 (5′- CATTTCGCTGCGTTCTT −3′), and 1005 (5′- TCGTTCAACACCTCATC −3′). The hybridized probes were immunodetected with anti-DIG-AP, Fab fragments (Roche) and were then visualized with the chemiluminescence substrate CDP-*Star*.

#### Immunoprecipitation

Cells expressing Rix1-5FLAG or Nsa1-5FLAG were harvested and washed using STOP buffer (150 mM NaCl, 50 mM NaF, 10 mM EDTA, 1mM NaN_3_ [pH 8.0]). Cell pellet was mixed with equal amount of buffer 1 (50 mM Tris-HCl (pH 7.5), 300 mM NaCl, 1.5 mM MgCl_2_, 0.3% NP-40, 1 mM PMSF, 1 mM DTT, 0.1 mM Na_3_VO_4_, protease inhibitor mix (Sigma)), and the mixture was beaten with glass beads using Fastprep bead-beater. Buffer 2 (50 mM Tris-HCl (pH 7.5), 1.5 mM MgCl_2_, 7.5% glycerol, 1 mM PMSF, 1 mM DTT, 0.1 mM Na_3_VO_4_, protease inhibitor mix (Sigma)) was added in volumes ∼2x the cell pellet, and the mixture was centrifuged (5k rpm, 2 min once, and 14k rpm, 10 min twice) to obtain supernatants (whole cell extracts). Immunoprecipitation was performed by incubating the whole cell extracts with anti-FLAG M2 monoclonal antibody (Sigma) and Dynabeads Protein G (Veritas) for 1 hr at 4°C. Whole cell extracts and immunoprecipitates were subjected to immunoblot analysis using mouse anti-HA monoclonal antibody 12CA5 (abcam), mouse anti-FLAG M2 monoclonal antibody (Sigma), or anti-GFP monoclonal antibody (Roche).

#### General Synthesis Information

##### Materials and instrumentation

Materials were purchased from Sigma-Aldrich and used without purification, unless otherwise noted. Reactions were run in capped round bottom flasks stirred with Teflon®-coated magnetic stir bars. Moisture- and air-sensitive reactions were performed in flame-dried round bottom flasks, fitted with rubber septa or glass gas adapters, under a positive pressure of nitrogen. Moisture- and air-sensitive liquids or solutions were transferred via nitrogen-flushed syringes. As necessary, solutions were deoxygenated by bubbling with nitrogen using a gas dispersion tube. Evaporation of solvents was accomplished by rotary evaporation using a Büchi rotary evaporator, equipped with a dry ice-acetone condenser, at 5-75 mm Hg at temperatures between 35°C and 50°C. Experiments were monitored by thin layer chromatography (TLC) or liquid chromatography mass spectrometry (LC-MS). The maintenance of 30°C to 150°C reaction temperatures was accomplished by the use of an oil bath. Products obtained as solids or high boiling oils were dried under vacuum (∼1 mmHg).

##### Chromatography

Compounds were purified using silica gel column chromatography, as necessary. Analytical TLC was performed using Whatman 250 micron aluminum backed UV F254 precoated silica gel flexible plates. Subsequent to elution, illumination at 254 nm allowed for visualization of UV absorbing materials. Staining with basic potassium permanganate solution allowed for further visualization.

##### Analytical Data

Proton nuclear magnetic resonance spectra (^1^H NMR) were recorded on Bruker DPX 400 MHz nuclear magnetic resonance spectrometer. Chemical shifts for ^1^H NMR spectra are reported as δ in units of parts per million (ppm) relative to tetramethylsilane (δ 0.0) using the residual solvent signal as an internal standard or tetramethylsilane itself: dimethylsulfoxide-*d*6 (δ 2.50, quintet) and deuterium oxide-*d*2 (δ 4.80, singlet). Multiplicities are given as: s (singlet), d (doublet), t (triplet), or m (multiplet). Coupling constants are reported as a *J* value in Hertz (Hz). The number of protons (n) for a given resonance is indicated by nH.

Liquid chromatography mass spectral analyses were obtained using a Waters MicroMassZQ mass spectrometer, with an electron spray ionization (ESI) probe, connected to a Waters 2795 HT Separation Module Alliance HT HPLC system running MassLynx (V4.0). The system used a Waters 996 Photodiode Array Detector set to 254 nm for peak detection, and a Symmetry® C18 (3.5 micron) 2.1 × 50 mm column for separation (mobile phase for positive mode: solvent A: water with 0.1% formic acid, solvent B: acetonitrile; mobile phase for negative mode: solvent A: water with 0.1% morpholine, solvent B: acetonitrile). Values are reported in units of mass to charge (*m/z*)

#### Synthesis of Rbin-1 analogs

**Figure:**
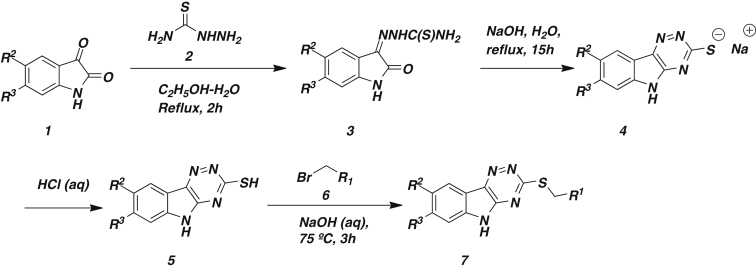

##### Scheme 1. Synthesis of Rbin-1 analogs

The synthesis method has been adapted from *J. Comb. Chem.* **2002,** *4,* 419-428. Briefly, a solution of thiosemicarbazide **2** (3.38 mmol, 1 equivalent) in boiling water was added with stirring to a solution of isatin or substituted isatin **1** (3.38 mmol, 1 equivalent) in boiling ethanol. The resulting mixture was refluxed for 2h to form a precipitate of thiosemicarbazone **3**, which was isolated by filtration and washed with ethanol. Thiosemicarbazone **3**, was then transferred to a solution of sodium hydroxide (3.38 mmol, 1equivalent) in water and refluxed overnight. The reaction mixture was then cooled to room temperature and the pH of the solution was adjusted to 3 with dilute aqueous HCl (1:3, v/v) to get precipitates of **5**. Triazinoindole **5** was then recrystallized from ethanol or washed with hot ethanol to get pure **5** and then dried in air.

Further elaboration of **5** was achieved by the addition of the alkyl bromide **6** (0.335 mmol, 0.67 equivalents) to a stirring solution of **5** (0.5 mmol, 1 equivalent) in 0.1 N NaOH (aqueous) and then refluxing the resulting mixture for 2h. The precipitated product was filtered, washed several times with water followed by hot ethanol to get pure **7**.

#### Synthesis of indole N-subsituted Rbin-1 analog 21

**Figure:**
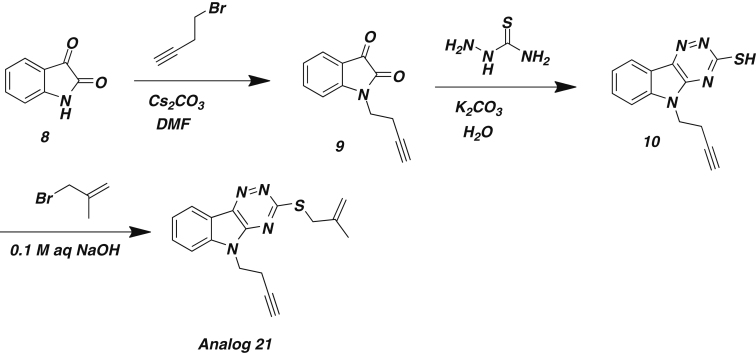

##### Scheme 2: Synthesis of analog 21

0.3 g Isatin **8** (0.2 mmol, 1eq) and 0.98 g of Cs_2_CO_3_ (0.03 mmol, 1.5 eq) were taken in a 25 ml two-neck round bottom flask and flushed with N_2_ for 30 min. 2 ml anhydrous DMF was added at 0°C while stirring. The resulting mixture was stirred for another 15 min at 0°C and warmed to room temperature and stirred for another hour. Then 4-bromo-1-butyne (0.06 μl, 3 eq) was added and stirred at 50°C overnight. After completion of the reaction 10 ml of 0.2 M HCl was added to the reaction mixture and poured into a separatory funnel. The product was extracted with 10 ml ethyl acetate (x 2) and dried over sodium sulfate. The solvent was evaporated using a rotary evaporator and the product **9** was dried overnight under high vacuum. The product **9** was then purified using silica gel column chromatography using 30% hexane/ethyl acetate.

Appropriate amount of *N*-homopropargyl isatin **9** (0.15 mmol, 1eq), semithiocarbazide (0.165 mmol, 1.1 eq), K_2_CO_3_ (0.15 mmol, 1eq) and 4 ml of water (500 μl of ethanol was added to help solubilizing isatin) were stirred and refluxed for 15 hr. The reaction mixture was then cooled to room temperature and acidified with 1:3 (v/v) HCl (aq) to pH ∼2. The precipitate was filtered and washed twice with water and once with ethanol. The product **10** was dried under vacuum.

13 μl of 3-Bromo-2-methyl propene (0.12 mmol, 1 eq) was added to a stirring solution of 0.03 g *N*-homopropargyl triazinoindole **10** in 0.1 M aqueous NaOH at refluxing temperatures. The resulting mixture was refluxed for 3 hr, then cooled to room temperature, filtered, and washed 3 times with water and once with ethanol. Product **analog 21** was dried under vacuum.

#### Characterization of the Rbin analogs

All chemical structures and naming of analogs are listed in Figure S2A.

Analog 1 or Rbin-1 (AP-5): ^1^H NMR (400 MHz, DMSO-d_6_) δ 1.84 (s, 3H), 3.98 (s, 2H), 4.91 (s, 1H), 5.1 (s, 1H), 7.42 (t, *J* = 7.38 Hz, 1H), 7.56 (d, *J* = 8.0 Hz, 1H), 7.68 (t, *J* = 7.54 Hz, 1H), 8.29 (d, *J* = 7.64 Hz, 1H). ESI-MS (m/z) [M+H]+ Calculated: 257.33, found: 257.57.
Analog 2 (ZC-9): ^1^H NMR (400 MHz, DMSO-d_6_) δ 3.96 (d, *J* = 6.68 Hz, 2H), 5.16 (d, *J* = 9.84 Hz, 1H), 5.39 (d, *J* = 16.8 Hz, 1H), 6.04 (m, 1H), 7.43 (t, *J* = 7.46 Hz, 1H), 7.52 (d, *J* = 8.0 Hz, 1H), 7.68 (t, *J* = 7.58 Hz, 1H), 8.30 (d, *J* = 8.0 Hz, 1H). ESI-MS (m/z) [M+H]+ Calculated: 243.30, found: 243.55.
Analog 3 (ZC11): ^1^H NMR (400 MHz, DMSO-d_6_) δ 1.03 (t, *J* = 7.28 Hz, 3H), 1.77 (m, 2H), 3.24 (t, *J* = 7.16 Hz, 2H), 7.43 (t, *J* = 7.44 Hz, 1H), 7.56 (d, *J* = 8.04 Hz, 1H), 7.68 (t, *J* = 7.6 Hz, 1H), 8.29 (d, *J* = 7.68 Hz, 1H). ESI-MS (m/z) [M+H]+ Calculated: 245.32, found: 245.57.
Analog 4 (AP-89): ^1^H NMR (400 MHz, DMSO-d_6_) δ 3.18 (s, 1H), 4.13 (s, 2H), 7.44 (t, *J* = 7.4 Hz, 1H), 7.58 (d, *J* = 8.04 Hz, 1H), 7.7 (t, *J* = 7.52 Hz, 1H), 8.32 (d, *J* = 7.64 Hz, 1H). ESI-MS (m/z) [M+H]+ Calculated: 241.28, found: 241.53.
Analog 5 (ZC-51): ^1^H NMR (400 MHz, DMSO-d_6_) δ 0.37 (m, *J* = 4.81 Hz, 2H), 0.58 (m, *J* = 5.9 Hz, 2H), 1.24 (m, 1H), 3.23 (d, *J* = 7.12 Hz, 2H), 7.43 (t, *J* = 7.43 Hz, 1H), 7.56 (d, *J* = 8.08 Hz, 1H), 7.68 (t, *J* = 7.48 Hz, 1H), 8.30 (d, *J* = 7.7 Hz, 1H). ESI-MS (m/z) [M+H]+ Calculated: 257.33, found: 257.59.
Analog 6 or Rbin-3 (AP-13): ^1^H NMR (400 MHz, DMSO-d_6_) δ 4.55 (s, 2H), 7.25 (t, *J* = 7.16 Hz, 1H), 7.32 (t, *J* = 7.22 Hz, 2H), 7.42 (t, *J* = 7.42 Hz, 1H), 7.51 (d, *J* = 7.24 Hz, 2H), 7.56 (d, *J* = 8.04 Hz, 1H), 7.68 (t, *J* = 7.5 Hz, 1H), 8.29 (d, *J* = 7.64 Hz, 1H). ESI-MS (m/z) [M+H]+ Calculated: 293.36, found: 293.62.
Analog 7 (AP-12): ^1^H NMR (400 MHz, DMSO-d_6_) δ 4.56 (s, 2H), 7.42 (t, *J* = 7.48 Hz, 1H), 7.52 (d, *J* = 4.96 Hz, 2H), 7.56 (d, *J* = 8.08 Hz, 1H), 7.68 (t, *J* = 7.62 Hz, 1H), 8.29 (d, *J* = 7.72 Hz, 1H), 8.50 (d, *J* = 3.72 Hz, 2H). ESI-MS (m/z) M+H]+ Calculated: 294.35, found: 294.59.
Analog 8 or Rbin-5 (ZC-10): ^1^H NMR (400 MHz, DMSO-d_6_) δ 4.74 (s, 2H), 7.43 (t, *J* = 7.54 Hz, 1H), 7.49 (t, *J* = 3.69 Hz, 2H), 7.58 (d, *J* = 8.08 Hz, 1H), 7.6 (m, 2H), 7.88 (d, *J* = 8.08 Hz, 3H), 8.04 (s, 1H), 8.30 (d, *J* = 7.6 Hz, 1H). ESI-MS (m/z) M+H]+ Calculated: 243.42, found: 243.65.
Analog 9 (ZC-5): ^1^H NMR (400 MHz, DMSO-d_6_) δ 3.73 (s, 3H), 4.53 (s, 2H), 6.83 (d, *J* = 7.2 Hz, 1H), 7.08 (d, *J* = 7.12 Hz, 2H), 7.24 (t, *J* = 7.9 Hz, 1H), 7.43 (t, *J* = 7.48 Hz, 1H), 7.58 (d, *J* = 8.04 Hz, 1H), 7.69 (t, *J* = 7.7 Hz, 1H), 8.31 (d, *J* = 7.6 Hz, 1H). ESI-MS (m/z) [M+H]+ Calculated: 323.39, found: 323.67.
Analog 10 (ZC-41): ^1^H NMR (400 MHz, DMSO-d_6_) δ 4.86 (s, 2H), 7.43 (t, *J* = 7.52 Hz, 1H), 7.56 (m, 2H), 7.7 (t, *J =* 7.72, 2H), 7.90 (d, *J* = 7.72 Hz, 1H), 8.05 (d, *J* = 8.12 Hz, 1H), 8.30 (d, *J* = 7.68 Hz, 1H). ESI-MS (m/z) [M+H]+ Calculated: 338.36, found: 338.61.
Analog 11 (ZC-7): ^1^H NMR (400 MHz, DMSO-d_6_) δ 4.69 (s, 2H), 7.44 (t, *J* = 7.6 Hz, 1H), 7.5 (d, *J* = 8.0 Hz, 1H), 7.69 (t, *J* = 7.6 Hz, 1H), 7.80 (d, *J* = 8.16 Hz, 2H), 8.17 (d, *J* = 8.16 Hz, 2H), 8.30 (d, *J* = 7.68 Hz, 1H). ESI-MS (m/z) M+H]+ Calculated: 338.36, found: 338.60.
Analog 12 or Rbin-4 (ZC-30): ^1^H NMR (400 MHz, DMSO-d_6_) δ 4.59 (s, 2H), 7.16 (t, *J* = 7.44 Hz, 1H), 7.22 (t, *J* = 9.28 Hz, 1H), 7.33 (m, 1H), 7.43 (t, *J* = 7.48 Hz, 1H), 7.58 (d, *J* = 8.08 Hz, 1H), 7.64 (t, *J* = 7.62 Hz, 1H), 7.69 (t, *J* = 7.64 Hz, 1H), 8.31 (d, *J* = 7.72 Hz, 1H). ESI-MS (m/z) [M+H]+ Calculated: 311.35, found: 311.57.
Analog 13 (AP-42): ^1^H NMR (400 MHz, DMSO-d_6_) δ 4.63 (s, 2H), 7.04 (t, *J* = 7.5 Hz, 1H), 7.36 (t, *J* = 7.4 Hz, 1H), 7.43 (t, *J* = 7.5, 1H), 7.58 (d, *J* = 8.04 Hz, 1H), 7.69 (t, *J* = 6.94 Hz, 2H), 7.90 (d, *J* = 7.68 Hz, 1H), 8.31 (d, *J* = 7.64 Hz, 1H). ESI-MS (m/z) [M+H]+ Calculated: 419.26, found: 419.48.
Analog 14 (AP-43): ^1^H NMR (400 MHz, DMSO-d_6_) δ 4.51 (s, 2H), 7.33 (d, *J* = 7.76 Hz, 2H), 7.43 (t, *J* = 7.46 Hz, 1H), 7.57 (d, *J* = 8.0 Hz, 1H), 7.68 (t, *J* = 8.98 Hz, 3H), 8.30 (d, *J* = 7.6 Hz, 1H). ESI-MS (m/z) [M+H]+ Calculated: 419.26, found: 419.55.
Analog 15 or Rbin-2 (ZC-32): ^1^H NMR (400 MHz, DMSO-d_6_) δ 1.84 (s, 3H), 3.98 (s, 2H), 4.91 (s, 1H), 5.12 (s, 1H), 7.58 (d, *J* = 8.28 Hz, 1H), 7.74 (s, 1H), 8.24 (d, *J* = 8.4 Hz, 1H). ESI-MS (m/z) [M]+ Calculated: 335.22, found: 335.45 and [M+2H]+ Calculated: 337.22, found: 337.48 (bromine pattern).
Analog 16 (ZC-33): ^1^H NMR (400 MHz, DMSO-d_6_) δ 4.56 (s, 2H), 7.26 (t, *J* = 7.24 Hz, 1H), 7.33 (t, *J* = 7.36 Hz, 2H), 7.51 (d, *J* = 7.36 Hz, 2H), 7.59 (d, *J* = 7.24 Hz, 1H), 7.75 (s, 1H), 8.24 (d, *J* = 8.32 Hz, 1H). ESI-MS (m/z) [M]+ Calculated: 371.26, found: 371.49 and [M+2H]+ Calculated: 373.26, found: 373.48 (bromine pattern).
Analog 17 (ZC-52): ^1^H NMR (400 MHz, DMSO-d_6_) δ 0.37 (d, *J =* 4.12 Hz, 2H), 0.58 (d, *J* = 7.2 Hz, 2H), 1.24 (m, 1H), 3.23 (d, *J* = 7.04 Hz, 2H), 7.58 (d, *J* = 8.12 Hz, 1H), 7.73 (s, 1H), 8.24 (d, *J* = 8.0 Hz, 1H). ESI-MS (m/z) [M]+ Calculated: 335.22, found: 335.52 and [M+2H]+ Calculated: 337.22, found: 337.55 (bromine pattern).
Analog 18 (AP-45): ^1^H NMR (400 MHz, DMSO-d_6_) δ 4.63 (s, 2H), 7.04 (t, *J* = 7.52 Hz, 1H), 7.36 (t, *J* = 7.4 Hz, 1H), 7.59 (d, *J* = 8.28 Hz, 1H), 7.7 (d, *J* = 7.56 Hz, 1H), 7.76 (s, 1H), 7.90 (d, *J* = 7.84 Hz, 1H), 8.25 (d, *J* = 8.2 Hz, 1H). ESI-MS (m/z) [M]+ Calculated: 497.15, found: 497.43 and [M+2H]+ Calculated: 499.15, found: 499.46 (bromine pattern).
Analog 19 (AP-46): ^1^H NMR (400 MHz, DMSO-d_6_) δ 4.5 (s, 2H), 7.33 (d, *J* = 8.08 Hz, 2H), 7.59 (d, *J* = 8.28 Hz, 1H), 7.67 (d, *J* = 8.16 Hz, 2H), 7.75 (s, 1H), 8.24 (d, *J* = 8.2, 1H). ESI-MS (m/z) [M]+ Calculated: 497.15, found: 497.44 and [M+2H]+ Calculated: 499.15, found: 499.45 (bromine pattern).
Analog 20 (AP-91): ^1^H NMR (400 MHz, DMSO-d_6_) δ 3.18 (s, 1H), 4.13 (s, 2H), 7.60 (d, *J* = 8.24 Hz, 1H), 7.74 (s, 1H), 8.26 (d, *J* = 8.28 Hz, 1H). ESI-MS (m/z) [M]+ Calculated: 319.18, found: 319.49 and [M+2H]+ Calculated: 321.18, found: 321.51 (bromine pattern).
Analog 21 or Rbin-6 (AP-38): ^1^H NMR (400 MHz, DMSO-d_6_) δ 2.78 (m, 3H), 4.0 (s, 2H), 4.55 (t, *J* = 6.66, 2H), 4.91 (s, 1H), 5.14 (s, 1H), 7.48 (t, *J* = 7.46 Hz, 1H), 7.75 (t, *J* = 7.44 Hz, 1H), 7.90 (d, *J* = 8,24 Hz, 1H), 8.34 (d, *J* = 7.64 Hz, 1H). ESI-MS (m/z) [M+H]+ Calculated: 309.40, found: 309.65.
Analog 22 or Rbin-7 (ZC-21): ^1^H NMR (DMSO) δ 1.85 (s, 3H), 3.99 (s, 2H), 4.91 (s, 1H), 5.12 (s, 1H), 7.42 (d, *J* = 8.4 Hz, 1H), 7.96 (d, *J* = 8.4 Hz, 1H), 8.59 (s, 1H). ESI-MS (m/z) [M+H]+ Calculated: 383.22, found: 383.51.
Analog 23 (ZC-25): ^1^H NMR (400 MHz, DMSO-d_6_) δ 4.56 (s, 2H), 7.25 (t, *J* = 7.04 Hz, 1H), 7.33 (t, *J* = 7.32 Hz, 2H), 7.42 (d, *J* = 8.4 Hz, 1H), 7.51 (d, *J =* 7.36 Hz, 2H), 7.96 (d, *J* = 8.56 Hz, 1H), 8.60 (s, 1H). ESI-MS (m/z) [M+H]+ Calculated: 419.26, found: 419.48.

#### Data reporting

Sample size was determined based on practical and experimental considerations. No statistical methods were used to predetermine sample size. The experiments were not randomized. The investigators were not blinded to allocation during experiments and outcome assessment.

### Quantification and Statistical Analysis

Unpaired t test was used for comparison between two groups whose variance is similar (calculated by F-test). Unpaired t test with Welch’s correction was used for comparison between two groups whose variance is different (calculated by F-test). Significance was considered when the P-value was less than 0.05.

## Author Contributions

S.A.K. and T.M.K. conceived and designed the project. S.A.K. performed most of the fission yeast experiments, except for construction and characterization of *mdn1-ts26* mutant (by Y.A.) and of Mdn1 ATPase mutants (by Y.A. and Y.K.). Z.C. and A.P. synthesized Rbin-1 and analogs. Z.C. purified recombinant Mdn1 (wild-type and mutant) and carried out all the biochemical assays. P.N. advised on the chemical screen and yeast genetics. S.A.K., Z.C., and T.M.K. wrote the manuscript with contributions from all other authors.

## Supplemental Information

Table S1. Fission Yeast Strains Used in This Study, Related to Star Methods and Figures 1, 2, 4, 5, 6, S1, S2, S3, S5, S6, and S7

Table S2. Plasmids Used in This Study, Related to Star Methods and Figures 2, 5, and S1
